# Mutation rates and selection on synonymous mutations in SARS-CoV-2

**DOI:** 10.1093/gbe/evab087

**Published:** 2021-04-24

**Authors:** Nicola De Maio, Conor R Walker, Yatish Turakhia, Robert Lanfear, Russell Corbett-Detig, Nick Goldman

**Affiliations:** 1 European Molecular Biology Laboratory, European Bioinformatics Institute, Wellcome Genome Campus, Hinxton, Cambridgeshire, CB10 1SD, UK; 2 Department of Genetics, University of Cambridge, Cambridge, CB2 3EH, UK; 3 Department of Biomolecular Engineering, University of California Santa Cruz, Santa Cruz, CA 95064, USA; 4 Genomics Institute, University of California Santa Cruz, Santa Cruz, CA 95064, USA; 5 Department of Ecology and Evolution, Research School of Biology, Australian National University, Canberra, ACT 2601, Australia

**Keywords:** SARS-CoV-2, COVID-19, Mutation, Selection, Sequencing, Viral genomics

## Abstract

The COVID-19 pandemic has seen an unprecedented response from the sequencing community. Leveraging the sequence data from more than 140,000 SARS-CoV-2 genomes, we study mutation rates and selective pressures affecting the virus. Understanding the processes and effects of mutation and selection has profound implications for the study of viral evolution, for vaccine design, and for the tracking of viral spread. We highlight and address some common genome sequence analysis pitfalls that can lead to inaccurate inference of mutation rates and selection, such as ignoring skews in the genetic code, not accounting for recurrent mutations, and assuming evolutionary equilibrium. We find that two particular mutation rates, G →U and C →U, are similarly elevated and considerably higher than all other mutation rates, causing the majority of mutations in the SARS-CoV-2 genome, and are possibly the result of APOBEC and ROS activity. These mutations also tend to occur many times at the same genome positions along the global SARS-CoV-2 phylogeny (i.e., they are very homoplasic). We observe an effect of genomic context on mutation rates, but the effect of the context is overall limited. While previous studies have suggested selection acting to decrease U content at synonymous sites, we bring forward evidence suggesting the opposite.

http://mc.manuscriptcentral.com/gbe

**Significance Statement:** Understanding how SARS-CoV-2 evolves within humans offers the chance of better estimating the effects of viral mutations, and of better reconstructing its transmission history, among other applications. Here, we investigate two of the main forces that shape SARS-CoV-2 evolution: the mutational process, and selection affecting synonymous mutations (mutations that do not involve amino acid changes that may impact viral proteins). We find that mutational forces in SARS-CoV-2 are highly variable across the genome. Also, by accounting for several pitfalls in the study of such data, we find new evidence for selection acting on synonymous mutations. These results help us understand SARS-CoV-2 evolution, design better vaccines, and improve our ability to track its transmission.

## 1 Introduction

The abundant and rapid availability of viral genomic data has had a profound effect on the response to the COVID-19 pandemic, from tracking and tracing of transmission (Zhang & Holmes, 2020; Deng et al., 2020; Dellicour et al.), to vaccine and drug development (Amanat & Krammer, 2020). Genomic SARS-CoV-2 data also allows us to investigate the evolutionary dynamics of the virus such as its mutational and selective pressures (Rice et al., 2020). Understanding the contribution of mutation and selection in shaping SARS-CoV-2 genome evolution is important, for example, for drug and vaccine development (Dearlove et al., 2020), for predicting variants of clinical and epidemiological importance (Korber et al., 2020; van Dorp et al., 2020b,a; Li et al., 2020; Cagliani et al., 2020), for understanding the biological mechanisms underlying viral genome evolution (such as recombination (Yi, 2020) and mutagenic immune system responses (Di Giorgio et al., 2020; Graudenzi et al., 2021; Mourier et al., 2020; Sadykov et al., 2020)), to improve the accuracy of phylogenetic approaches for epidemiological applications (Duchene et al., 2020; Phelan et al., 2020; Ramazzotti et al., 2020) and for inferring its origin (Nielsen et al., 2020).

When deciphering genome evolution, one has to disentangle the effect of mutation and selection affecting the emergence and spread of genetic variants. It has been observed by several studies that SARS-CoV-2 presents a very skewed mutational spectrum, with most observed genetic variation resulting from C$\rightarrow$U mutations (Simmonds, 2020; Rice et al., 2020; De Maio et al., 2020a; Turakhia et al., 2020a). It is important to account for these mutational skews when inferring selection, since recurrent mutations can generate a phylogenetic signal that can be confused with positive selection when using phylogenetic methods (e.g. Yang, 2007; Pond & Muse, 2005). Such strong mutational skews, if unaccounted for, can also cause errors in phylogenetic tree inference (De Maio et al., 2020a; Turakhia et al., 2020a).

Here, we identify common pitfalls when analysing SARS-CoV-2 genomic data, and we present an alternative, robust approach for identifying the contribution of mutation and selection in SARS-CoV-2 evolution. We confirm that the C$\rightarrow$U mutation rate is very high in SARS-CoV-2, in particular within the context http://mc.manuscriptcentral.com/gbe

U$\underline{\text{C}}$G→U$\underline{\text{U}}$G, putatively as the result of APOBEC ("Apolipoprotein B mRNA Editing Catalytic Polypeptide-like") proteins activity. However, the majority of C→U mutations occur outside of the U$\underline{\text{C}}$G context. Secondly, and in contrast to most other studies, we find that the G→U mutation rate is nearly as high (about 97%) as C→U. The reason why this has not been noted in most other studies is because they did not control for the biases in the genetic code. In fact, the genetic code tends to be more robust to transitions (e.g. C→U) than transversions (e.g. G→U), see Freeland & Hurst (1998); Goldman (1993). This has a large effect on inferred mutation rates also because most of the SARS-CoV-2 genome is composed of coding sequence. This causes G→U mutations to be underrepresented among the observed genetic variation, despite its high mutation rate. We control for this issue by focusing on synonymous mutations, which are subject to more moderate levels of selective pressure than non-synonymous mutations (Cuevas et al., 2012). Finally, we investigate selection acting on synonymous variants. We find evidence contrary to previous claims of selection against U content (Rice et al., 2020).

### 2 New Approaches

Estimating accurate mutation rates is essential for understanding the evolutionary and immunological pressures acting on the virus, as well as to infer accurate phylogenies and for detecting selection. One of the main aims of our work is the estimation of mutation rates in SARS-CoV-2, in particular while trying to control for the effects of selection, which can affect the spread of certain types of mutation, and therefore decrease or increase our chances of detecting them.

State of the art inference of evolutionary rates often entails inferring a substitution rate matrix describing sequence evolution along a phylogenetic tree using maximum likelihood (Yang, 1994; Whelan et al., 2001). However, the large number of SARS-CoV-2 sequences available makes this kind of approach challenging from a computational perspective (Morel et al.; Hodcroft et al., 2021), in particular if we are interested in more complex models that describe codon evolution and context dependency (Siepel & Haussler, 2004; Kosiol et al., 2007; De Maio et al., 2013a).

Previous studies investigating mutation rates in SARS-CoV-2 (see e.g. Rice et al., 2020; Simmonds, 2020) have used an approach more typical for within-population and cancer data, that is, based on counting the number of genome positions at which alternative alleles are observed (i.e. the numbers of different types of SNPs, see e.g. Harris & Pritchard, 2017; Alexandrov et al., 2013). This approach works well when the number of mutations that occurred is small relative to the number of genome positions considered. However, when the same mutation events occur multiple times at the same position on different branches of the phylogenetic tree (as is the case for SARS-CoV-2, De Maio et al., 2020a; Turakhia et al., 2020a; van Dorp et al., 2020a), this approach can underestimate the most elevated mutation rates, since multiple mutation events can end up conflated and counted as a single variant allele.

http://mc.manuscriptcentral.com/gbe

The approach we propose here consists instead of, first, inferring a maximum likelihood phylogenetic tree for the considered SARS-CoV-2 genomes. Then, we infer a mutational history on this tree for each position of the genome using parsimony. This gives us, for each given mutation type (for example, synonymous mutations from nucleotide A to C) an estimated number of mutation events. We then normalize this number of mutation events, dividing it by the number of mutation "possibilities" (see Equation 1 in Section 5.2). These possibilities represent the number of sites in the reference genome in which a mutation of a certain type is possible. Synonymous, nonsynonymous, nonsense and non-coding possibilities are counted separately. At each position, multiple mutation events of the same type are possible along the phylogeny, so the inferred number of mutation events of a certain type can be larger than the number of mutation possibilities for the same type. These normalized counts represent our estimates of relative mutation rates, and in particular normalized synonymous mutation counts represent our estimates of neutral mutation rates.

In order to obtain multiple independent estimates, and to correct for the contribution of possible issues in our sequences, we separate mutation counts in 3 bins according to the number of descendants of each mutation: (i) those with only one descendant tip ("singletons"); (ii) those with $\geq 2$ and $\leq 4$ descendants ("2-4 descendants"); and (iii) those with $> 4$ descendants ("$> 4$ descendants"). The main rationale for this is that errors in genome sequences are expected to mostly cause apparent mutation events with only 1 or very few descendants. While we do notice sequence artefacts at higher frequency among genome sequences, we can more easily detect these, and we mask them from our alignment following De Maio et al. (2020a,b). These artefacts, in fact, tend to be associated with only one or a few sequencing laboratories, while generating a characteristic signal of homoplasy in the phylogenetic tree (De Maio et al., 2020a; Turakhia et al., 2020a).

For completeness, we also show results from the alternative approach used in previous studies estimating mutation rates, based on counting the number of alignment columns at which alternative alleles are observed. We note again, however, that due to possibly multiple mutation events of the same type occurring at the same site in different lineages, this approach is expected to underestimate the number of mutation events, in particular for recurring mutations. Similarly to before, we define three classes of variant alleles: (i) those present in any number of sequences; (ii) those present in at least two sequences; (iii) those present in at least five sequences.

Another issue that needs to be considered when estimating mutation rates is that different mutations can have different effects on the ability of the virus to replicate and transmit. In this manuscript, we mainly focus on synonymous mutations, as they are sufficiently abundant in our dataset to allow reliable estimates of mutation rates, and as they are expected to usually have a more limited effect on the viral fitness (Cuevas et al., 2012), therefore providing reduced biases in the inference of neutral mutation rates.

Later in the manuscript we describe a method to investigate possible mild but consistent fitness effects of synonymous mutations in SARS-CoV-2. This http://mc.manuscriptcentral.com/gbe method is based on the comparison of ratios of estimated numbers of mutations with different numbers of descendants. The principle behind this method is that negative selection tends to decrease the frequency of new mutations, while positive selection tends to increase it. So, for example, if we want to compare the fitness of synonymous C→U mutations versus A→C ones, we can compare the ratio of high-frequency vs low-frequency synonymous C→U mutations, to the same ratio for synonymous A→C mutations. If the former is significantly higher, we take this as evidence that synonymous C→U mutations have typically higher fitness than A→C ones.

More details on our approaches are given in the Results and Material and Methods sections below.

## 3 Results

### 3.1 Neutral mutation rates in SARS-CoV-2

Here, we want to estimate the underlying mutability of different nucleotides, in a way that is as unbiased as possible with regards to how these mutations might affect the ability of of the virus to replicate and spread. To do this, firstly, we mostly focus on synonymous mutations, since a synonymous mutation is expected to affect, on average, the fitness of the virus considerably less than a nonsynonymous mutation (Cuevas et al., 2012). Secondly, we only consider new SARS-CoV-2 mutations observed within the human host population, and ignore long-term divergence (between-species substitutions) which is expected to be more affected by selective forces (McDonald & Kreitman, 1991) and more subject to the issues of rate changes (see e.g. Panchin & Panchin, 2020) and saturation (se e.g. Duchêne et al., 2014). While we cannot exclude that selection still affects some of the patterns observed below, for example making some types of synonymous mutations more lethal for the virus than others, we tried to reduce these biases as much as possible as follows:

First, to put our results in the perspective of previous studies, we looked at numbers of sites with alternative alleles. When looking at patterns across the whole genome, it appears that all transitions (C→U, U→C, A→G and G→A) are quite common, as well as G→U mutations; note however that A and U bases have more opportunities to mutate as they are more common in the genome (Figure 1A). If we focus only on possible synonymous mutations (which we expect to be less likely affected by strong selection), we see that C→U and G→U synonymous alternative variants are present at the vast majority of sites at which such variants are possible (Figure 1B). This means that sites at which synonymous C→U and G→U mutations might have occurred are possibly saturated with such mutations. In fact, using our phylogenetic approach to estimate numbers of mutation events of each type at each genome position, we clearly see that C→U and G→U are the most frequent mutations (Figure 1C), despite having fewer opportunities to occur due to GC content being lower than AT content. Focusing again on synonymous mutations, we can see that, while we http://mc.manuscriptcentral.com/gbe don't infer more overall G→U mutations than U→C or A→G ones, given the very low number of sites at which synonymous G→U mutations are possible, G→U and C→U are the synonymous mutations that occur proportionally more often (Figure 1D). We also observe similar patterns for non-coding, non-synonymous and 4-fold degenerate sites (Figure S1). These observations suggest that the G→U and C→U underlying neutral mutation rates are considerably higher than all others.

Our results further suggest that inferring mutation rates from counting the number of sites with variants (the counts in Figure 1A-B, and as in e.g. Rice et al. (2020); Simmonds (2020)) can lead to underestimating the G→U and C→U synonymous mutation rates. This is because sites at which such mutations are possible are probably often saturated (multiple mutation events of exactly the same type have occurred along the phylogeny). However, saturation in previous studies was probably not as extreme as here since we investigate a considerably larger number of genomes (147,137 vs for example 14,599 in Rice et al. (2020) and 865 in Simmonds (2020)). Another difference of our approach from most previous studies (but similar to Rice et al., 2020), is that we aim to disentangle the contribution of selection acting on the amino acid sequence from the underlying mutation rates, and to do this we separate synonymous and nonsynonymous mutations. This has a considerable further effect on the inference of the G→U mutation rates, because there are only a few sites at which a G→U mutation is synonymous, and so most possible G→U mutations are probably under significant purifying selection; skews in the genetic code (and in particular the fact that the genetic code tends to be more robust to transitions than transversions Freeland & Hurst, 1998; Goldman, 1993) are probably an important factor here. Furthermore, G is less frequent in the SARS-CoV-2 genome than U, in particular at 4-fold degenerate sites (sites where any 1-base mutation is synonymous) with a frequency of 6.5%, compared to frequencies of A 28.9%, C 11.4%, and U 50.9%; this causes a further under-representation of observed G→U mutations relative to the underlying G→U mutation rate.

A confirmation that many synonymous G sites are saturated with mutations can be seen in Figures 2 and S2. C→U and G→U mutations are by far the most homoplasic (the same mutation occurs more than once along the phylogeny), especially at synonymous and non-coding positions. This is unlikely to be the result of positive selection favouring these mutations, or of phylogenetic tree inference or parsimony mutation inference errors (since this effect is only observed in C→U and G→U mutations) and seems instead the result of underlying relatively high neutral C→U and G→U mutation rates.

We also mostly find individual C→U or G→U mutations among the most homoplasic mutations of the genome: 55 out of 59 total mutation events that occur more than 60 times at the same position are C→U or G→U. Here we ignore sites that are so homoplasic as to probably cause issues in phylogenetic inference (Turakhia et al., 2020a; De Maio et al., 2020a); these sites were masked before any analysis here, and are namely the G→U mutation at position 11083, and the C→U mutations at positions 16887 and 21575. These three sites appear as the most mutable in the genome. However, the next 3 most homoplasic http://mc.manuscriptcentral.com/gbe

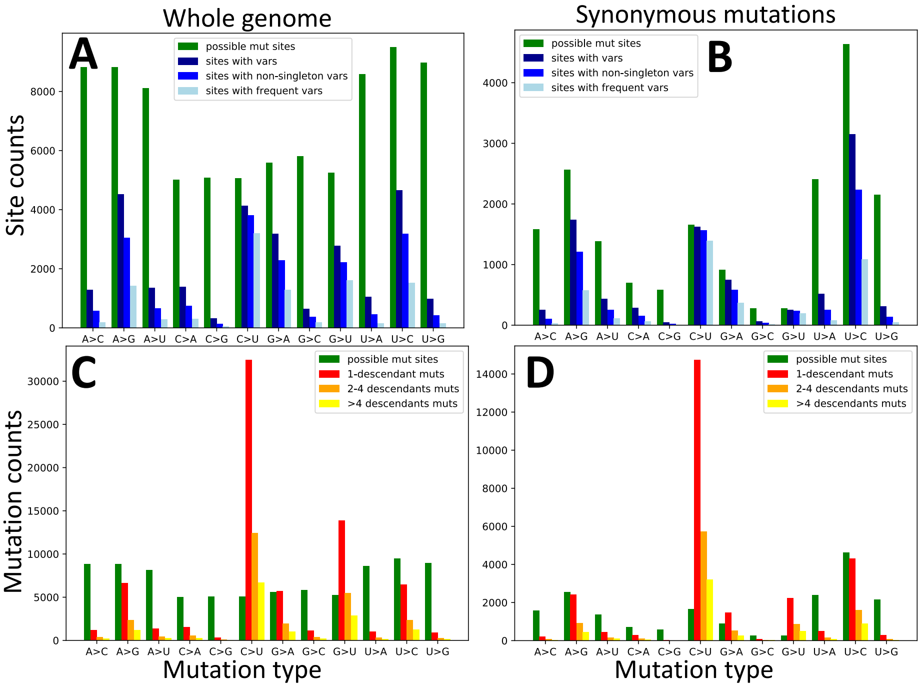

Figure 1: **Numbers of possible mutations, observed mutations, and sites with alternative alleles**. On the X axes are the 12 distinct types of mutation events, A→C, A→G, etc. In green we always show the number of genome positions at which the considered mutation type is possible. In **A,C** we consider all possible mutations, while in **B,D** we consider only synonymous mutations. In **A,B** we show, on the Y axis, the numbers of sites with alternative alleles in the alignment (blue color hues). Note that Y axis scales differ among plots. In dark blue we show the number of all sites with alternative variants of the given type; in blue, we only show the number of such sites at which the alternative variant is present in at least 2 sequences; in light blue, only sites at which the considered alternative allele is present in at least 5 sequences. By definition, in plots **A-B** green bars are necessarily taller than all blue ones. In **C-D** we show, in red, orange and yellow, the numbers of mutation events inferred with parsimony on our phylogeny. In red we show the number of mutation events of the considered type with exactly one descendant; in orange the number of these mutations with at least 2 but less than 5 descendants; in yellow, those with at least 5 descendants. Mutation possibilities (green) can be fewer than inferred mutations events (red, orange and yellow in plots **C-D**) for certain types of mutations since the same mutation event can be inferred multiple times at the same site in different parts of the phylogenetic tree.

http://mc.manuscriptcentral.com/gbe

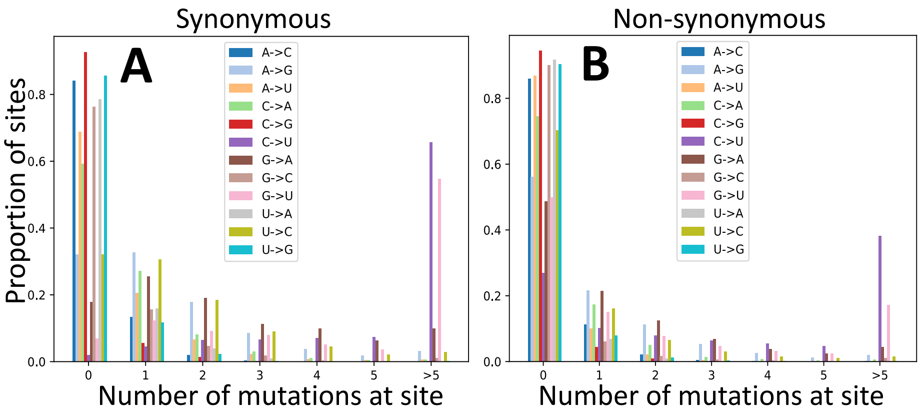

Figure 2: **Re-occurrence of mutation events at the same sites**. Proportion of sites (Y axis) where a given mutation (color, see legends) appears a certain number of times (X axis) along the phylogeny. **A** synonymous sites; **B** non-synonymous sites.

mutations of the genome that we identify here are neither C→U nor G→U mutations. The most recurrent one is the A→G nonsynonymous (K→R) mutation at position 10323 which we inferred to have occurred 138 times, with a total of 1187 descendants, found in 3'→5' context CTTAAGCTTAAGGTTGATACA. The second is a A→G nonsynonymous (K→R) mutation at position 21137, which occurred 130 times with a total of 472 descendants, found in context ATACAACAAAAGCTAGCTCTT. The third is a T→C synonymous mutation at position 27384 which occurred 119 times, with a total of 808 descendants, found in context TGGAGATTGATTAAACGAACA. We suspect that these 3 mutations are the results of frequent ADAR ("Adenosine Deaminase Acting on RNA") activity, considering also their context. For example, the first two are A→G mutations with a G downstream and an A upstream (see Porath et al., 2014; Ko et al., 2012). The third site is probably affected by ADAR acting on the negative strand, since its T→C mutation is the reverse complement of the typical A→G ADAR pattern. The fourth highly homoplasic mutation we found that is not G→U or C→U is the 11th most common in the genome, a G→A nonsynonymous (G→S) mutation at position 1820, with a total of 409 descendants and in context AGCTAAAAAAGGTGCCTGGAA.

One of the principal aims of our work is to estimate mutation rates while controlling the effect of selection acting on the amino acid sequence, and trying to account as much as possible for other issues such as homoplasic mutations. To do this, firstly, we focus on synonymous mutations, which are expected to be less subject to selective constraints than nonsynonymous ones, while being much more abundant than non-coding mutations. Secondly, we use inferred counts of mutation events (using parsimony inference along our phylogenetic tree, see http://mc.manuscriptcentral.com/gbe

| | to A | to C | to G | to U |
|---|---|---|---|---|
| from A | | 0.039 | 0.310 | 0.123 |
| from C | 0.140 | | 0.022 | 3.028 |
| from G | 0.747 | 0.113 | | 2.953 |
| from U | 0.056 | 0.261 | 0.036 | |

Table 1: **Mutation rates estimated from 4-fold degenerate sites**. Only mutation events with more than 1 descendant have been considered here. Rates have been normalized as typically done in phylogenetics, the normalizing constant being the sum of the rates multiplied by the frequency of the ancestral allele: $\sum_{a,b} q_{a,b}\pi(a)$, where $a, b$ are nucleotides, $q_{a,b}$ is the original unnormalized rate from allele $a$ to $b$, and $\pi(a)$ is the frequency of $a$ at 4-fold degenerate sites.

Methods); this accounts for the saturation of mutation events at more mutable positions, as discussed before. Thirdly, we separate mutation counts according to the number of observed descendants of each mutation - this allows us to have independent estimates, and to have estimates that do not rely on inferred mutation events with one descendant, which might be enriched in sequencing errors or RNA degradation (in the case errors RNA degradation would be present in our alignment). Fourthly, we normalize mutation counts by the number of mutation possibilities (see Equation 1 in Section 5.2), so to account for the fact that certain mutation types (for example synonymous G→U mutations) are possible at fewer sites than other mutation types. For this last point, we divide the number of inferred mutations of a certain type (e.g., red, orange and yellow bars in Figure 1D) by the number of sites at which such mutations are possible (e.g., green bars in Figure 1D), always considering the reference genome as the mutational ancestral background. We find that the G→U transversion mutation rate is similar to C→U transition rate (Figures 3 and S3), and that they are both considerably higher than all other rates (about 4 times higher than the next highest rate, G→A, see Table 1). We confirm that all other transitions, U→C, G→A and A→G, have lower rates, but higher than all remaining transversions. In particular, the mutational process seems highly asymmetrical and strand-asymmetrical, with mutation rate G→U being 82.0 times higher than U→G and 21.1 times higher than C→A (Table 1). This strand-asymmetry is likely the result of ROS ("Reactive Oxygen Species") and APOBEC activity on single-strand RNA (Graudenzi et al., 2021; Mourier et al., 2020).

Our results differ markedly from those studies that either estimated mutation rates by comparing numbers of sites with alternative alleles, did not divide these counts by the numbers of opportunities for such mutations, and/or did not control for the biases in the genetic code by separating synonymous and nonsynonymous mutations (e.g. Wang et al., 2020; Kosuge et al., 2020). Our results are instead more consistent with those of studies that did take some of these steps and highlighted similarly high C→U and G→U mutation rates in SARS-CoV-2 (e.g. Rice et al., 2020). When we use an approach more similar http://mc.manuscriptcentral.com/gbe

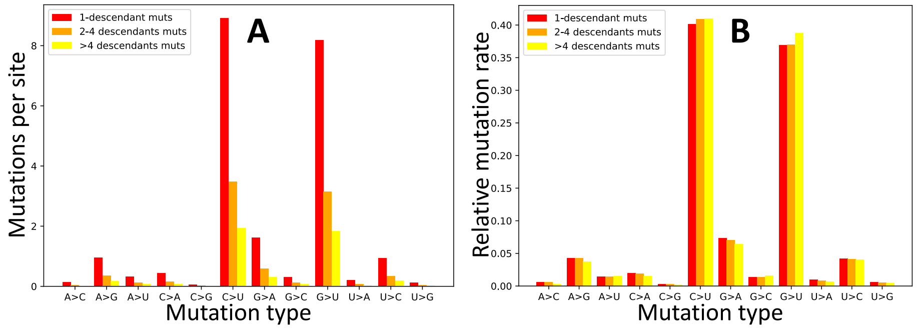

Figure 3: **Estimated synonymous mutation rates in SARS-CoV-2**. To estimate synonymous mutation rates in SARS-CoV-2 we used the counts of inferred synonymous mutation events (see Fig 1D) normalized by the numbers of reference genome sites at which such mutations might have occurred. On the X axis are the 12 distinct types of mutation events, A→C, A→G, etc. In red, orange and yellow we show respectively rates obtained from counts of mutation events with 1 descendant, more than 1 but less than 5 descendant, and 5 or more descendants. **A** Mutation rates represented as average numbers of mutation events inferred per site at which such mutation type is possible. **B** Relative mutation rates (the sum of all bars of one specific color is 1.0).

to classical maximum likelihood substitution rate inference (e.g. Yang, 2007, see Section 5.2) we find almost the same results (Figure S4), suggesting that our approximations have little effect on our inferred rates. While elevated C→U mutation rate in SARS-CoV-2 has been frequently observed and usually attributed to the effects of APOBEC activity, the elevated G→U has been discussed much less, but it has been usually suggested to be the result of the activity of reactive oxygen species (ROS), see Rice et al. (2020); Mourier et al. (2020). The number of descendants of mutation events seems to have little impact on our inferred relative mutation rates (Figure 3B); this suggests that phenomena like RNA degradation or sequencing errors, which are expected to overwhelmingly result in inferred mutation events with one descendant, do not considerably affect our mutation rate estimates.

### 3.2 Context dependencies

One of the observations that suggests APOBEC activity being the leading cause of the elevated C→U mutation rate in SARS-CoV-2 is that nucleotide context seems to affect the C→U mutation rate in a way that is consistent with the action of some of the human APOBECs (Mourier et al., 2020). Here we study the effect of neighbouring base context on C→U mutability. We divide the set of possible and observed C→U mutations in classes based on the nucleotide context (5' to 3' preceding and following base). Even though we focus only on C→U

http://mc.manuscriptcentral.com/gbe

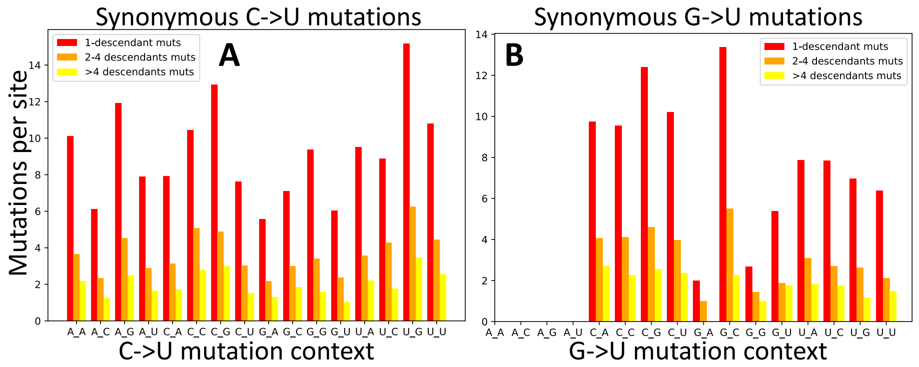

Figure 4: **C→U and G→U synonymous mutation rates in different base contexts**. Here mutation rates are calculated as in Figure 3A. **A** C→U mutations. **B** G→U mutations. The X axis shows the context of the considered mutation (for example, in **A**, A_G represents the trinucleotide ACG and its synonymous mutation rate into trinucleotide AUG). Colors are as in Figure 3.

mutations, it is still important to separate synonymous and nonsynonymous mutations, since different contexts can lead to synonymous or nonsynonymous C→U mutations with different proportions. Additionally, different contexts can be present with different frequencies in the SARS-CoV-2 genome. Focusing on synonymous mutations, and using the normalization procedure outlined above, we find that UCG→UUG appears to be the most mutable context (Figures 4A and S5). Note that this pattern is not observed if one considers unnormalized mutation counts, particularly those from nonsynonymous mutations (Figure S6).

This confirms previous results which suggested an elevated UCG→UUG mutation rate (Kosuge et al., 2020; Sadykov et al., 2020; Rice et al., 2020), attributed to either the context-specificity of APOBEC mutational targets, or to selection against CpG dinucleotides (Mourier et al., 2020). We discuss selection in the next section. Here, we note that while we found a signal of context affecting mutation rates in SARS-CoV-2, and while at least part of this observation is consistent with some of the known sequence targets of APOBEC, overall, C→U mutations occurring in UCG or more generally UC→UU context represent only part (and a very small part for UCG) of all C→U mutations in SARS-CoV-2 (Figure S1) and contribute to only a small part of the C→U relative hypermutability.

Regarding longer-range sequence context (nearest 5 bases in each direction) we observe that U in the 2 bases preceding a C seems to have a C→U mutagenic effect, while G in the previous 2 bases and C in the following 2 bases seem to reduce the mutation rate (Figure S7).

Given the relatively high G→U mutation rate, we performed a similar analysis of context effects on G→U mutations. However, despite the elevated mutation rate, G→U synonymous mutations are quite rare (Figure S8) and so estimates http://mc.manuscriptcentral.com/gbe of context-dependent rates are expected to have substantial uncertainty. Additionally, some contexts are not possible for G→U synonymous mutations, for example A<u>G</u>→A<u>U</u> mutations are never synonymous (Figure S8). The effect of sequence context on G→U mutation rate appears overall limited, although a G being preceded or followed by a C (C<u>G</u> and <u>G</u>C contexts) seems to have a higher G→U mutation rate (Figures 4B and S9).

### 3.3 Testing for selection at synonymous sites using mutation frequencies

Previous authors have discussed the effect of selection on synonymous mutations in SARS-CoV-2. For example, the elevated mutation rate in CpG context has been interpreted as a sign of selection against CpG content in SARS-CoV-2 in order to avoid zinc finger antiviral proteins (ZAP), see Mourier et al. (2020). This is consistent with similar evidence found from depleted CpG content in other coronaviruses (Woo et al., 2007). However, evidence from CpG content and substitution rates can be confounded by skewed mutation rates and mutational contexts like the ones due to APOBEC activity, which might be the leading cause of mutation in SARS-COV-2.

To overcome this limitation, and in order to disentangle the contribution of mutation and selection over the synonymous evolution of SARS-CoV-2, Rice et al. (2020) compared equilibrium frequencies inferred from SARS-CoV-2 mutation rates to observed nucleotide and dinucleotide frequencies. Since selection tends to raise the frequency of favourable alleles in a population, and to decrease the frequency of deleterious alleles, if a certain nucleotide is advantageous over another at synonymous sites, for example, if C is advantageous over U, it is expected that relatively more U→C mutations will reach fixation (completely replace the ancestral allele in the population) than C→U mutations. This means that, in the long term, there would be fewer U nucleotides in the genome than expected based on the mutation rates estimated from observed genetic variation within the population. Rice et al. (2020) observed that U nucleotides are less common at 4-fold degenerate sites in SARS-CoV-2 than expected from SARS-CoV-2 genetic variation within humans, and concluded that there is ongoing selection against U nucleotides in SARS-CoV-2. With a similar analysis, they also concluded that there is no ongoing selection against CpG content in SARS-CoV-2, despite opposite prior expectations (see Mourier et al., 2020; Woo et al., 2007).

Rice et al. (2020) assumed that the evolutionary process is stationary, that is, that mutation rates and selective pressures did not change recently (for example, since the introduction of SARS-CoV-2 into humans). This assumption is however debatable. Due to the recent host shift and to the fact that many mutations in SARS-CoV-2 seem to be the consequence of host immune system activity, a recent significant change in mutation rates and selective pressure in SARS-CoV-2 (associated with its introduction to humans) is likely. In fact, studies have suggested that the current G→U mutation rate of SARS-CoV-2 in humans, for example, is much higher than in its reservoir hosts (Panchin & Panchin, 2020; http://mc.manuscriptcentral.com/gbe

Sapoval et al., 2021). Specifically, Panchin & Panchin (2020) estimated that the G→U mutation rate in SARS-CoV-2 increased 9-fold with its introduction in humans, possibly due to difference in ROS activity between hosts.

The U content at 4-fold degenerate sites in the SARS-CoV-2 reference genome is 50.9%. This value is expected to represent very closely the U content at 4-fold degenerate sites just before the introduction of the virus to the human populations. The approach of Rice et al. (2020) consists of comparing this value with the equilibrium U content at 4-fold degenerate sites estimated from mutation rates inferred from variant sites within the human host population; a higher equilibrium U content is interpreted as evidence of selection, on average, against U content. To highlight some of the issues with this approach, we consider how a shift in mutation rates associated with the human host might affect equilibrium U content. We consider 4 different sets of mutation rates, each of which leads to different equilibrium nucleotide frequencies (which we estimated using the python package discreteMarkovChain v0.22 `https://pypi.org/project/discreteMarkovChain/`). First, we consider the mutation rates at 4-fold degenerate sites estimated following Rice et al. (2020) on our dataset (inferred from the numbers of sites with variant alleles, as in Figure 1B); secondly, we use the same rates but decreasing the G→U mutation rate 9-fold (to mimic the putative reservoir mutation rate estimated in Panchin & Panchin, 2020); thirdly, we use our estimates of mutation rates as in Table 1; lastly, we use the rates as in Table 1 but again decreasing the G→U mutation rate 9-fold. Using these 4 sets of mutation rates we obtain equilibrium U content at 4-fold degenerate sites of respectively 65.5%, 52.6%, 77.4% and 64.9%. Therefore, it is clear that changes in mutation rates associated with host shift can have dramatic effects on equilibrium frequencies, which can affect the inference of selective pressure using this approach. Furthermore, this analysis is based on the assumption that selective pressures on U content are the same in humans and in the reservoir host, which is not obvious.

To address these issues, we propose an alternative approach to test for selection acting on synonymous mutations. Since selection increases the frequency of favourable alleles and decreases the frequency of deleterious alleles within a population, we expect selection to change the relative proportion of alleles at different population frequencies. The McDonald-Kreitman test (McDonald & Kreitman, 1991), for example, compares numbers of synonymous and nonsynonymous within-species polymorphisms and between-species differences; within this framework, purifying selection is expected to decrease the number of nonsynonymous between-species substitutions more than the number of nonsynonymous within-species polymorphisms.

However, the emergence of SARS-CoV-2 in humans is relatively recent, too recent for variants (and especially a statistically sufficient number of variants) to have reached fixation within the human population, making this type of test inapplicable. Instead, we use an alternative version in which we compare low-frequency mutations against high-frequency ones. While our aim is to focus on comparing different types of synonymous mutations, we first apply this approach to compare synonymous and nonsynonymous mutations, so as to make http://mc.manuscriptcentral.com/gbe

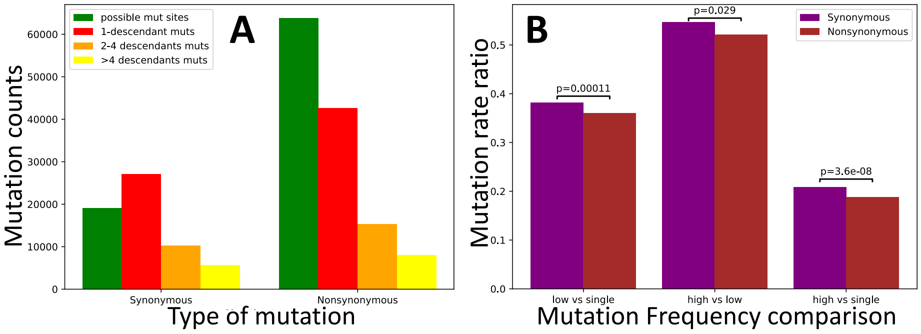

Figure 5: **Evidence of selection affecting the population frequency of synonymous vs nonsynonymous mutations**. Counts and rate ratios of SARS-CoV-2 synonymous and non-synonymous mutations at different frequencies in the human population. **A** counts of possible mutations (green), singleton mutations (red), mutations with $> 1$ and $\leq 4$ descendants (orange), and mutations with $> 4$ descendants (yellow). **B** ratios of higher vs. lower frequency mutation rates. In the absence of selection, ratios should not be significantly different between the classes of synonymous and nonsynonymous mutations. Instead, we measure a significant deviation in each comparison, with nonsynonymous mutations being relatively depleted of high frequency mutations. We calculated p-values using the chi2_contingency function of the Scipy.stats package (Virtanen et al., 2020). On the X axis, "single" refers to mutations with 1 descendant, "low" to mutations with 2-4 descendants, and "high" to mutations with >4 descendants. For example, "high vs single" refers to the comparison of rate of mutations with >4 descendants versus the rate of mutations with 1 descendant.

the similarity to the McDonald-Kreitman test more apparent. Nonsynonymous mutations in SARS-CoV-2 appear significantly shifted towards lower frequencies (Figure 5), consistent with the expectation that purifying selection tends to decrease the frequencies of nonsynonymous mutations more than the frequencies of synonymous mutations.

Next, we focused on the hypothesis that U variants at synonymous sites are on average mildly deleterious (Rice et al., 2020). This time, not all of our comparisons are significant, but those that are, consistently suggest that selection favours U variants (Figure 6), not the opposite. As we discussed before, the difference with the estimate from Rice et al. (2020) probably lies in the assumption of stationarity in SARS-CoV-2 evolution (although we also used a larger dataset and a different approach to estimate mutation rates). It's hard to draw clear conclusions regarding individual genes, as, expectedly, we lose statistical power when analyzing each gene individually, in particular for the shorter genes (Figures S10 and S11).

A possible explanation for the pattern we observe is that many mutations http://mc.manuscriptcentral.com/gbe

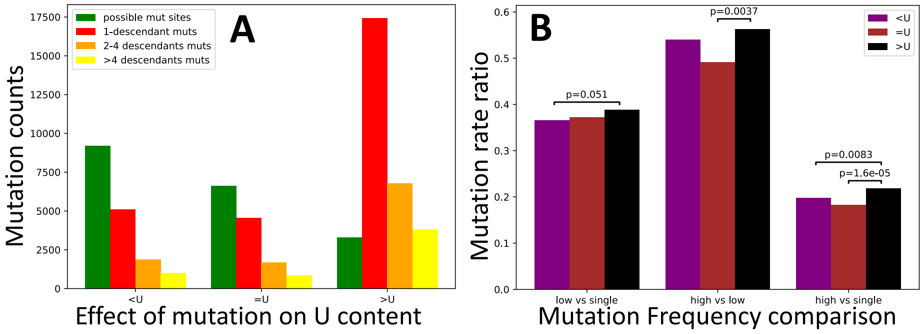

Figure 6: **Test of selection affecting U content at synonymous sites**. Values are the same as in Figure 5, but this time we focus on synonymous mutations that decrease U content ("<U"), increase it (">U"), or leave it unaltered ("=U"). Only p-values below 0.1 are shown.

increasing U content are C→U or G→U, and therefore may sometimes reduce CpG content. Selection against CpG content has already been suggested in coronaviruses (Woo et al., 2007), and is expected due to ZAP activity (Mourier et al., 2020). However, using our approach the evidence in support of this hypothesis is not significant (Figure S12). Similarly, when investigating possible selection affecting GC content, only one of the comparisons is significant (Figure S13).

## 4 Discussion

In this study, we investigated mutation rates and selection at synonymous sites in SARS-CoV-2. We used a different methodology than previous studies, so as to exploit more than 147,000 SARS-CoV-2 genome sequences while trying to avoid inaccuracies due to mutation saturation. Our approach also controls for the fact that different types of mutations can have, on average, different fitness due to their impact on the amino acid sequence and due to skews in the genetic code. In fact, we focus on synonymous mutations, which are expected to be subject to lower selective pressure than nonsynonymus mutations. Also, we did not assume equilibrium in SARS-CoV-2 genome evolution, which is unlikely given the recent shift of host the virus underwent. We also investigated some possible selective pressure affecting synonymus mutations.

We found that two mutation rates, C→U and G→U, are similar to each other and much higher than all others mutation rates, leading to extremely frequent homoplasies. This also means that the SARS-CoV-2 mutation process is very far from being symmetric or strand-symmetric; it is also considerably far from equilibrium. We found some sites with extremely high non-C→U non-G→U mutation rates that are consistent with targeted ADAR activity.

http://mc.manuscriptcentral.com/gbe

A consequence of these findings is that popular and efficient phylogenetic substitution models such as JC69 (Jukes et al., 1969), HKY85 (Hasegawa et al., 1985), or GY94 (Goldman & Yang, 1994) might be inappropriate with SARS-CoV-2 data and might cause biases in the inference of phylogenetic trees (Turakhia et al., 2020a), selection (van Dorp et al., 2020a; Graudenzi et al., 2021) and recombination (Yi, 2020) due to the neutral high recurrence of certain mutations. It is therefore probably important to adopt phylogenetic substitution model that can account for elevated C→U and G→U rates, such as the non-reversible UNREST model (Yang, 1994), or otherwise to control for the skews in the mutational process (see e.g. Graudenzi et al., 2021). In our experience, we have found the UNREST model to often cause numerical instability in current phylogenetic packages that allow it (data not shown here). In the future, if we want to alleviate these biases, it will be important to implement such more general substitution models more broadly and in a more numerically stable way.

When investigating selection at synonymous sites possibly affecting CpG or GC content, we found mostly non-significant patterns. We also investigated the possibility of selection against U content in SARS-CoV-2 (Rice et al., 2020), but found evidence in the opposite direction. While further analyses will be needed to establish with confidence what kind of selection acts on SARS-CoV-2 synonymous mutations, we suggest that inference based on assumption of genome equilibrium can be biased by changes in mutation rates and selective pressures associated with host shift. While we tried to account for possible biases as much as possible, our methods still have some limitations. First of all, our inference of mutation events is based on a prior phylogenetic inference, but tree inference from SARS-CoV-2 data can be unreliable, in particular regarding low-level branches of the phylogeny (those closer to the samples and further from the root), in part due to the low genetic diversity among sequences, but more importantly due to highly homoplasic mutations and sequencing errors (Morel et al.; Turakhia et al., 2020a); Removing extremely homoplasic sites and putative errors can be of help, but, as we have shown, the mutational spectrum in SARS-CoV-2 is highly skewed, and so removing all homoplasic sites would result in removing most C→U and G→U synonymous mutations, which in turn would cause the loss of a large part of the available phylogenetic signal. As mentioned above, our phylogenetic inference might also have been negatively affected by the choice of substitution models; however, currently, more realistic models like UNREST are either not implemented or are numerically unstable in sufficiently efficient phylogenetic packages such as Minh et al. (2020); Price et al. (2010); Kozlov et al. (2019). In this study we tried not to rely excessively on individual inferences of mutation events, but rather focused on general patterns averaged over many sites and clades, which we think should provide robust inference despite the fact the inference of individual mutation events might not be reliable. However, a potential bias that might affect our result derives from the fact that some sites are very homoplasic, and our phylogenetic inference might lead to an over-parsimonious inference of their mutational history. This, in turn, might lead us to underestimate their mutation rate and overestimate their number of descendant tips per mutation events. In the future, a Bayesian http://mc.manuscriptcentral.com/gbe phylogenetic approach might be useful to assess and possibly resolve this issue and assess its impact on our inference of selective pressure; however so far Bayesian phylogenetic inference has proved prohibitive with datasets of this size.

To further investigate and disentangle selective and mutational forces in SARS-CoV-2, it would be very promising to combine an analysis of between-patients and within-patient SARS-CoV-2 genetic variation. In a similar way as selection is expected to decrease the frequency of deleterious SARS-CoV-2 alleles at the human population level, the same is true at the within-host levels, as selection is expected to act on within-host deleterious mutations and often prevent them from reaching high frequency and transmit further on. While within-patient genetic diversity data can indeed be very informative of the SARS-CoV-2 evolutionary patterns (Di Giorgio et al., 2020; Graudenzi et al., 2021; Kuipers et al., 2020), it is important to consider that such data is also more prone to sequencing, read processing, and RNA degradation issues. These issues cause some errors in consensus sequences (De Maio et al., 2020a; Turakhia et al., 2020a) but they are expected to be even more problematic at the level of detected within-host variation. Indeed, so far there is reason to be cautious when interpreting these data (De Maio et al., 2020a), especially with specific datasets (see `https://virological.org/t/gained-stops-in-data-from-the-peter-doherty-institute-for-infection-and-immunity/486`). Given reliable data regarding evolution at different levels (e.g. within a patient, between patients, and between hosts), it would be interesting to combine these sources of information to improve estimates of selective pressures (see e.g. Wilson et al., 2011; De Maio et al., 2013b).

Our methodology to detect selection could also be improved by using the full site frequency spectrum of mutations, instead of categorizing mutations into frequency classes (see e.g. Zeng & Charlesworth, 2009; Clemente & Vogl, 2012). However, it is important to consider that complex epidemiological dynamics and sampling biases in SARS-CoV-2 mean that it is hard to interpret the shape of any individual site frequency spectrum in terms of the effects of mutation and selection. We infer mutation and selection patterns by comparing properties of site frequency spectra associated with different mutation types. This approach should be robust to the effects of variable population dynamics and sampling biases, since these forces are expected to affect in the same way site frequency spectra associated with different mutation types.

While we only applied our methods to SARS-CoV-2 genome data, the same approaches could be used for variation data from other viruses. Other studies have suggested that mutation rates in SARS-CoV-2 are quite different from those in SARS-CoV and MERS, with SARS-CoV-2 showing, for example, higher G$\rightarrow$U rates (Panchin & Panchin, 2020; Sapoval et al., 2021). It would be of interest to test if these estimates would change after controlling for saturation and selection at the amino acid level.

In the future, we hope to extend our current approach to also study insertions and deletions (indels): their frequency, recurrence and possible fitness effects (see e.g. Kemp et al., 2020). However, we expect that incorporating indels in our approach will be challenging since standard phylogenetic approaches rarely http://mc.manuscriptcentral.com/gbe model indels.

## 5 Materials and Methods

### 5.1 Data collection and phylogenetic inference

Full details and reproducible code for the construction of the global tree of SARS-CoV-2 samples are available in the 13/11/20 release of Lanfear (2020). To summarise, this code creates a global phylogeny of all available samples from the GISAID data repository as follows.

First, all sequences marked as 'complete' and 'high coverage' submitted up to 13/11/20 were downloaded from GISAID. Sequences with known issues from previous analyses were then removed from this database (details are in the excluded_sequences.tsv file at DOI `10.5281/zenodo.3958883`).

Second, a global alignment was created by aligning every sequence individually to the NC_045512.2 accession from NCBI, using MAFFT v 7.471 (Katoh & Standley, 2013), faSplit (`http://hgdownload.soe.ucsc.edu/admin/exe/`), faSomeRecords (`https://github.com/ENCODE-DCC/kentUtils`), and GNU parallel (Tange et al., 2011). This approach aligns each sequence individually to the reference, then joins them into a global alignment by ignoring insertions relative to the reference.

Third, sites that are likely to be dominated by sequencing error (Turakhia et al., 2020a) are masked from the alignment using faSplit, seqmagick (`https://seqmagick.readthedocs.io/en/latest/`), and GNU parallel, sequences shorter than 28KB or with more than 1000 ambiguities are removed from the alignment using esl-alimanip (`hmmer.org`), and subsequently sites that are $> 50\%$ gaps are removed (after converting N's to gaps) using esl-alimask.

Fourth, the global phylogeny was estimated using IQ-TREE 2 (Minh et al., 2020) and FastTree 2 (Price et al., 2010) (v2.1.10 compiled with double precision) in two stages. First, new sequences added to GISAID between 11/11/20 and 13/11/20 were added to the phylogeny inferred on 11/11/20 using Maximum Parsimony placement in IQ-TREE 2. This produces a starting tree of all sequences available on 13/11/20. Second, the starting tree was optimised using FastTree 2 with 2 rounds of Subtree Pruning and Regrafting (SPR) using moves of length 1000 under a minimum evolution optimisation regime, and the tree was then further optimised using multiple rounds of Maximum Likelihood Nearest Neighbour Interchange (NNI) moves until no further improvement to the tree could be achieved using NNI. The resulting tree was rooted with the reference sequence (NC_045512.2 / MN908947.3 / Wuhan/Hu-1) using nw_reroot (Junier & Zdobnov, 2010).

From the resulting tree, we removed sequences on very long branches using TreeShrink (Mai & Mirarab, 2018), using the default false positive tolerance rate $q = 0.05$ to identify such branches. These sequences are likely to be either of poor quality and/or poorly aligned, so rather unreliable to interpret in a phylogeny with such limited variation. We cannot exclude that some lineages http://mc.manuscriptcentral.com/gbe with true series of mutations (e.g. like lineage B.1.1.7 Tang et al., 2020) are excluded this way, but only if these lineages have very few sequences in our dataset; furthermore, the exclusion of certain lineages is not expected to lead to biases in our analyses. The final tree and its related alignment contains 147,137 SARS-CoV-2 genomes.

### 5.2 Estimation of mutation rates

To separate mutation events into different categories, each position of the reference genome (NC_045512.2 / MN908947.3 / Wuhan/Hu-1, see `https://www.ncbi.nlm.nih.gov/nuccore/MN908947`) was classified as coding or non-coding. Given the very short evolutionary distances considered, the choice of reference genome is unlikely to significantly affect our inferences; in fact, changing the reference genome would only invert the direction of a handful of mutation events. Start and Stop codons were not considered in the following analysis. The first and last 100bp of the genome, in addition to sites marked as problematic in `https://github.com/W-L/ProblematicSites_SARS-CoV2/blob/master/problematic_sites_sarsCov2.vcf` (De Maio et al., 2020a; Turakhia et al., 2020a), were also not considered here. We counted "possibilities" of mutations based on the reference genome: for example a non-coding C allele in the reference genome represents 3 possibilities for non-coding mutations (C→A, C→G and C→U). For coding sites, we split synonymous, non-synonymous and nonsense mutation possibilities into separate counts. Similarly, we also used the reference genome to define the number of possible mutations within each genetic context. Sites that were masked in the alignment were still used here to define the genetic context of possible mutations at neighbouring non-masked sites.

We then inferred a mutational history with parsimony over our global maximum likelihood tree, as described in Turakhia et al. (2020a). Given the very short branches of the SARS-CoV-2 phylogeny (median 0 and mean 0.46 mutations per phylogenetic branch per genome) uncertainty in ancestral state reconstruction conditional on the phylogenetic tree is negligible. The software for this analysis is available from `https://github.com/yatisht/strain_phylogenetics`. Given this inferred mutational history, for each site $x$ we count all the mutation events $N_x^{j,d}$ inferred from the reference allele $r_x$ at position $x$ to any alternative allele $j \neq r_x$, and with $i$ descendants, excluding those that are descendants of further downstream mutations at the same site. Note that we do not count mutation events that modify a non-reference allele. For example, if at a U position of the genome we have a U→C mutation, and in one of the descendants of this mutation event we have a C→A mutation at the exact same position, then we count the first U→C mutation but not the second C→A; this is done to aid the normalization of mutation counts, see below. The inferred mutation events excluded this way are 2,040 out of a total 116,945 (1.7% of the total). At each site and for each mutation type, we kept counts of three different classes of mutations: (i) those with only one descendant tip ("singletons"); (ii) those with $\geq 2$ and $\leq 4$ descendants ("2-4 descendants"); and (iii) those with $> 4$ descendants ("$> 4$ descendants"). This is done to help rule out the possibility that low-frequency sequence errors might cause many artefactual mutations and bias our rate inference; in fact, such artefactual mutations are expected to tendentially have only very few descendants. Sites with frequent sequencing errors are already addressed by masking the corresponding alignment columns, following De Maio et al. (2020a); Turakhia et al. (2020a). Numbers of mutations and mutation possibilities observed across the SARS-CoV-2 genome are given in Figures 1 and S1. Note that the number of observed mutations is often higher than the number of mutation possibilities. For example, a reference 4-fold degenerate site with a reference A allele counts as one A→C, one A→G and one A→U synonymous mutation possibility. However, an A→C mutation event can occur multiple times along the phylogeny at this site, possibly resulting in multiple A→C synonymous mutations observed. When classifying mutation events into different categories, we assume that mutations happen in the genetic background of the reference genome. This might be inaccurate in some cases, but given the overall low level of divergence the effect of this approximation is expected to be very limited. In fact, given that genomes in our dataset have on average about 11.9 differences from the reference, and, for simplicity, assuming that these changes are uniformly distributed across the genome (of length ≈29900 bp) and across samples, then the probability a site is mutated is $\approx 11.9/29900 \approx 0.04\%$, and the probability that any of two sites neighbouring a mutation event (for example those determining if the mutation is synonymous) is different from the reference (and therefore that the reference context misrepresents the mutation context) is $\approx 0.08\%$. This is likely to be an over-estimate since mutation events happen on internal branches of the tree, which are on average more similar to the reference genome than the sampled genomes themselves, and so will have an average number of differences from the reference genome below the values used here of 11.9. To estimate relative mutation rates, we divide the number of mutations inferred for a certain class by the number of its mutation possibilities. For example, the relative rate of synonymous mutations $r_{a,b}$ from allele $a$ to allele $b$ with more than 4 descendants is estimated as:

$$\frac{\sum_{x\in\{y|r_y=a\}}\sum_{d>4} I(x,b)N_x^{b,d}}{\sum_{x\in\{y|r_y=a\}} I(x,b)}, \tag{1}$$

where $\{y|r_y = a\}$ is the set of genome positions where the reference allele is $a$, and where $I(x, b)$ is an indicator function that returns 1 if changing the reference at position $x$ into allele $b$ is a synonymous mutation, and 0 otherwise.

The low mutation rate observed in SARS-CoV-2, and in particular the low number of mutation events per phylogenetic branch, makes parsimony an efficient and reliable approach to infer mutational histories (Turakhia et al., 2020b). However, phylogenetic inference from large SARS-CoV-2 datasets is difficult due to elevated computational demand and phylogenetic uncertainty (Morel et al.), and we cannot exclude the presence of errors in our phylogenetic tree, and therefore in our mutational history.

To test the effects of our approximations on the estimated mutation rates, we also used a different approach, more similar to the classical maximum likelihood http://mc.manuscriptcentral.com/gbe inference of substitution rates in phylogenetics (see e.g. Yang, 2007), to estimate synonymous and nonsynonymous mutation rates. To do this, we used the same phylogenetic tree and inferred mutational history as above. This time, however, we count all mutations, no matter their background, as long as they don't happen within masked regions of the alignment or from a stop codon. This removes our approximation of only considering mutation events from the reference allele. Also, this time we keep track of the context of each mutation events using the reconstructed ancestral genome at the considered mutation event, removing the assumption that the context of a mutation is the same as the reference genome at that position. Finally, when counting mutation possibilities, we consider each position in the reconstructed ancestral genomes, and weigh them using the corresponding branch lengths below them, therefore accounting for the fact that the genome evolves along the phylogeny when estimating mutation possibilities. The estimates obtained with this approach are very similar to those from our main approach described above (see Figure S4).

Finally, we also consider another alternative approach to estimating mutation rates, based on counting the number of alignment columns at which alternative alleles are observed. We classify three classes of variant allele: (i) those present in any number of sequences; (ii) those present in at least two sequences; (iii) those present in at least five sequences. Numbers of different types of variable sites found in our alignment are given in Figures 1 and S1. This last approach is similar to those used by previous studies os SARS-CoV-2 mutation rates, and we use it for comparison with our new main approach.

## 6 Supplementary Material

The Supplementary File contains all supplementary figures.

## 7 Data Availability

The code and data used for this project are available at `https://github.com/NicolaDM/mutSelSarsCov2`. The genome sequences fall under the restrictions of the GISAID (`https://www.gisaid.org/`) terms of use and can only be downloaded from GISAID by registered users.

## 8 Acknowledgments

NG, CW and NDM were supported by the European Molecular Biology Laboratory (EMBL). R.C.-D. was supported by R35GM128932 and by an Alfred P. Sloan foundation fellowship. R.L. is funded by Australian Research Council grant DP200103151, and by a Chan-Zuckerberg Initiative grant. We are very grateful to GISAID and all the groups who shared their sequencing data. A full list of acknowledgments is available from `https://github.com/roblanf/sarscov2phylo/tree/master/acknowledgements`.

http://mc.manuscriptcentral.com/gbe
