# Mutation Rates and Selection on Synonymous Mutations in SARS-CoV-2

**DOI:** 10.1093/gbe/evab087

**Published:** 2021-04-24

**Authors:** Nicola De Maio, Conor R Walker, Yatish Turakhia, Robert Lanfear, Russell Corbett-Detig, Nick Goldman

**Affiliations:** 1 European Molecular Biology Laboratory, European Bioinformatics Institute, Cambridgeshire, United Kingdom; 2 Department of Genetics, University of Cambridge, United Kingdom; 3 Department of Biomolecular Engineering, University of California, Santa Cruz, California, USA; 4 Genomics Institute, University of California, Santa Cruz, California, USA; 5 Department of Ecology and Evolution, Research School of Biology, Australian National University, Canberra, ACT, Australia

**Keywords:** SARS-CoV-2, COVID-19, mutation, selection, sequencing, viral genomics

## Abstract

The COVID-19 pandemic has seen an unprecedented response from the sequencing community. Leveraging the sequence data from more than 140,000 SARS-CoV-2 genomes, we study mutation rates and selective pressures affecting the virus. Understanding the processes and effects of mutation and selection has profound implications for the study of viral evolution, for vaccine design, and for the tracking of viral spread. We highlight and address some common genome sequence analysis pitfalls that can lead to inaccurate inference of mutation rates and selection, such as ignoring skews in the genetic code, not accounting for recurrent mutations, and assuming evolutionary equilibrium. We find that two particular mutation rates, G →U and C →U, are similarly elevated and considerably higher than all other mutation rates, causing the majority of mutations in the SARS-CoV-2 genome, and are possibly the result of APOBEC and ROS activity. These mutations also tend to occur many times at the same genome positions along the global SARS-CoV-2 phylogeny (i.e., they are very homoplasic). We observe an effect of genomic context on mutation rates, but the effect of the context is overall limited. Although previous studies have suggested selection acting to decrease U content at synonymous sites, we bring forward evidence suggesting the opposite.

## Introduction

The abundant and rapid availability of viral genomic data has had a profound effect on the response to the COVID-19 pandemic, from tracking and tracing of transmission (Deng et al. 2020; Zhang and Holmes 2020; Dellicour et al. 2021), to vaccine and drug development (Amanat and Krammer 2020). Genomic SARS-CoV-2 data also allows us to investigate the evolutionary dynamics of the virus such as its mutational and selective pressures (Rice et al. 2020). Understanding the contribution of mutation and selection in shaping SARS-CoV-2 genome evolution is important, for example, for drug and vaccine development (Dearlove et al. 2020), for predicting variants of clinical and epidemiological importance (Cagliani et al. 2020; Korber et al. 2020; Li et al. 2020; van Dorp et al. 2020a, 2020b), for understanding the biological mechanisms underlying viral genome evolution (such as recombination [Yi 2020] and mutagenic immune system responses [Di Giorgio et al. 2020; Graudenzi et al. 2021; Mourier et al. 2021; Sadykov et al. 2020]), to improve the accuracy of phylogenetic approaches for epidemiological applications (Duchene et al. 2020; Phelan et al. 2020; Ramazzotti et al. 2021), and for inferring its origin (Wang et al. 2021).

When deciphering genome evolution, one has to disentangle the effect of mutation and selection affecting the emergence and spread of genetic variants. It has been observed by several studies that SARS-CoV-2 presents a very skewed mutational spectrum, with most observed genetic variation resulting from C →U mutations (Rice et al. 2020; Simmonds 2020; De Maio et al. 2020a; Turakhia et al. 2020a). It is important to account for these mutational skews when inferring selection, since recurrent mutations can generate a phylogenetic signal that can be confused with positive selection when using phylogenetic methods (e.g., Pond and Muse 2005; Yang 2007). Such strong mutational skews, if unaccounted for, can also cause errors in phylogenetic tree inference (De Maio et al. 2020a; Turakhia et al. 2020a).

Here, we identify common pitfalls when analyzing SARS-CoV-2 genomic data, and we present an alternative, robust approach for identifying the contribution of mutation and selection in SARS-CoV-2 evolution. We confirm that the C →U mutation rate is very high in SARS-CoV-2, in particular within the context UCG →UUG, putatively as the result of APOBEC (“Apolipoprotein B mRNA Editing Catalytic Polypeptide-like”) proteins activity. However, the majority of C →U mutations occur outside of the UCG context. Second, and in contrast to most other studies, we find that the G →U mutation rate is nearly as high (about 97%) as C →U. The reason why this has not been noted in most other studies is because they did not control for the biases in the genetic code. In fact, the genetic code tends to be more robust to transitions (e.g., C →U) than transversions (e.g., G →U), see Freeland and Hurst (1998) and Goldman (1993). This has a large effect on inferred mutation rates also because most of the SARS-CoV-2 genome is composed of coding sequence. This causes G →U mutations to be underrepresented among the observed genetic variation, despite its high mutation rate. We control for this issue by focusing on synonymous mutations, which are subject to more moderate levels of selective pressure than nonsynonymous mutations (Cuevas et al. 2012). Finally, we investigate selection acting on synonymous variants. We find evidence contrary to previous claims of selection against U content (Rice et al. 2020).

## New Approaches

Estimating accurate mutation rates is essential for understanding the evolutionary and immunological pressures acting on the virus, as well as to infer accurate phylogenies and for detecting selection. One of the main aims of our work is the estimation of mutation rates in SARS-CoV-2, in particular while trying to control for the effects of selection, which can affect the spread of certain types of mutation, and therefore decrease or increase our chances of detecting them.

State-of-the-art inference of evolutionary rates often entails inferring a substitution rate matrix describing sequence evolution along a phylogenetic tree using maximum likelihood (Yang 1994; Whelan et al. 2001). However, the large number of SARS-CoV-2 sequences available makes this kind of approach challenging from a computational perspective (Morel et al. 2020; Hodcroft et al. 2021), in particular if we are interested in more complex models that describe codon evolution and context dependency (Siepel and Haussler 2004; Kosiol et al. 2007; De Maio, Holmes, et al. 2013a).

Previous studies investigating mutation rates in SARS-CoV-2 (see, e.g., Rice et al. 2020; Simmonds 2020) have used an approach more typical for within-population and cancer data, that is, based on counting the number of genome positions at which alternative alleles are observed (i.e., the numbers of different types of SNPs, see, e.g., Harris and Pritchard 2017; Alexandrov et al. 2013). This approach works well when the number of mutations that occurred is small relative to the number of genome positions considered. However, when the same mutation events occur multiple times at the same position on different branches of the phylogenetic tree (as is the case for SARS-CoV-2, De Maio et al. 2020a; Turakhia et al. 2020a; van Dorp et al. 2020a), this approach can underestimate the most elevated mutation rates, since multiple mutation events can end up conflated and counted as a single variant allele.

The approach we propose here consists instead of, first, inferring a maximum-likelihood phylogenetic tree for the considered SARS-CoV-2 genomes. Then, we infer a mutational history on this tree for each position of the genome using parsimony. This gives us, for each given mutation type (e.g., synonymous mutations from nucleotide A to C) an estimated number of mutation events. We then normalize this number of mutation events, dividing it by the number of mutation “possibilities” (see eq. 1). These possibilities represent the number of sites in the reference genome in which a mutation of a certain type is possible. Synonymous, nonsynonymous, nonsense, and noncoding possibilities are counted separately. At each position, multiple mutation events of the same type are possible along the phylogeny, so the inferred number of mutation events of a certain type can be larger than the number of mutation possibilities for the same type. These normalized counts represent our estimates of relative mutation rates, and in particular normalized synonymous mutation counts represent our estimates of neutral mutation rates.

In order to obtain multiple independent estimates, and to correct for the contribution of possible issues in our sequences, we separate mutation counts in three bins according to the number of descendants of each mutation: 1) those with only one descendant tip (“singletons”); 2) those with ≥2 and ≤4 descendants (“2–4 descendants”); and 3) those with >4 descendants (“>4 descendants”). The main rationale for this is that errors in genome sequences are expected to mostly cause apparent mutation events with only 1 or very few descendants. Although we do notice sequence artefacts at higher frequency among genome sequences, we can more easily detect these, and we mask them from our alignment following De Maio et al. (2020a, 2020b). These artefacts, in fact, tend to be associated with only one or a few sequencing laboratories, whereas generating a characteristic signal of homoplasy in the phylogenetic tree (De Maio et al. 2020a; Turakhia et al. 2020a).

For completeness, we also show results from the alternative approach used in previous studies estimating mutation rates, based on counting the number of alignment columns at which alternative alleles are observed. We note again, however, that due to possibly multiple mutation events of the same type occurring at the same site in different lineages, this approach is expected to underestimate the number of mutation events, in particular for recurring mutations. Similarly to before, we define three classes of variant alleles: 1) those present in any number of sequences; 2) those present in at least two sequences; 3) those present in at least five sequences.

Another issue that needs to be considered when estimating mutation rates is that different mutations can have different effects on the ability of the virus to replicate and transmit. In this manuscript, we mainly focus on synonymous mutations, as they are sufficiently abundant in our data set to allow reliable estimates of mutation rates, and as they are expected to usually have a more limited effect on the viral fitness (Cuevas et al. 2012), therefore providing reduced biases in the inference of neutral mutation rates.

Later in the manuscript, we describe a method to investigate possible mild but consistent fitness effects of synonymous mutations in SARS-CoV-2. This method is based on the comparison of ratios of estimated numbers of mutations with different numbers of descendants. The principle behind this method is that negative selection tends to decrease the frequency of new mutations, whereas positive selection tends to increase it. So, for example, if we want to compare the fitness of synonymous C →U mutations versus A →C ones, we can compare the ratio of high-frequency versus low-frequency synonymous C →U mutations, to the same ratio for synonymous A →C mutations. If the former is significantly higher, we take this as evidence that synonymous C →U mutations have typically higher fitness than A →C ones.

More details on our approaches are given in the Material and Methods and Results sections.

## Materials and Methods

### Data Collection and Phylogenetic Inference

Full details and reproducible code for the construction of the global tree of SARS-CoV-2 samples are available in the November 13, 2020 release of Lanfear (2020). To summarize, this code creates a global phylogeny of all available samples from the GISAID data repository as follows.

First, all sequences marked as “complete” and “high coverage” submitted up to November 13, 2020 were downloaded from GISAID. Sequences with known issues from previous analyses were then removed from this database (details are in the excluded_sequences.tsv file at DOI 10.5281/zenodo.3958883).

Second, a global alignment was created by aligning every sequence individually to the NC_045512.2 accession from NCBI, using MAFFT v 7.471 (Katoh and Standley 2013), faSplit (http://hgdownload.soe.ucsc.edu/admin/exe/, last accessed May 9, 2021), faSomeRecords (https://github.com/ENCODE-DCC/kentUtils, last accessed May 9, 2021), and GNU parallel (Tange et al. 2011). This approach aligns each sequence individually to the reference, then joins them into a global alignment by ignoring insertions relative to the reference.

Third, sites that are likely to be dominated by sequencing error (Turakhia et al. 2020a) are masked from the alignment using faSplit, seqmagick (https://seqmagick.readthedocs.io/en/latest/, last accessed May 9, 2021), and GNU parallel, sequences shorter than 28KB or with more than 1,000 ambiguities are removed from the alignment using esl-alimanip (hmmer.org), and subsequently sites that are >50% gaps are removed (after converting N’s to gaps) using esl-alimask.

Fourth, the global phylogeny was estimated using IQ-TREE 2 (Minh et al. 2020) and FastTree 2 (Price et al. 2010) (v2.1.10 compiled with double precision) in two stages. First, new sequences added to GISAID between November 11, 2020 and November 13, 2020 were added to the phylogeny inferred on November 11, 2020 using Maximum Parsimony placement in IQ-TREE 2. This produces a starting tree of all sequences available on November 13, 2020. Second, the starting tree was optimized using FastTree 2 with 2 rounds of subtree pruning and regrafting using moves of length 1,000 under a minimum evolution optimization regime, and the tree was then further optimized using multiple rounds of maximum-likelihood nearest-neighbor interchange (NNI) moves until no further improvement to the tree could be achieved using NNI. The resulting tree was rooted with the reference sequence (NC_045512.2/MN908947.3/Wuhan/Hu-1) using nw_reroot (Junier and Zdobnov 2010).

From the resulting tree, we removed sequences on very long branches using TreeShrink (Mai and Mirarab 2018), using the default false positive tolerance rate *q *=* *0.05 to identify such branches. These sequences are likely to be either of poor quality and/or poorly aligned, so rather unreliable to interpret in a phylogeny with such limited variation. We cannot exclude that some lineages with true series of mutations (e.g., like lineage B.1.1.7, Tang et al. 2021) are excluded this way, but only if these lineages have very few sequences in our data set; furthermore, the exclusion of certain lineages is not expected to lead to biases in our analyses. The final tree and its related alignment contains 147,137 SARS-CoV-2 genomes.

### Estimation of Mutation Rates

To separate mutation events into different categories, each position of the reference genome (NC_045512.2/MN908947.3/Wuhan/Hu-1, see https://www.ncbi.nlm.nih.gov/nuccore/MN908947, last accessed May 9, 2021) was classified as coding or noncoding. Given the very short evolutionary distances considered, the choice of reference genome is unlikely to significantly affect our inferences; in fact, changing the reference genome would only invert the direction of a handful of mutation events. Start and stop codons were not considered in the following analysis. The first and last 100 bp of the genome, in addition to sites marked as problematic in https://github.com/W-L/ProblematicSites_SARS-CoV2/blob/master/problematic_sites_sarsCov2.vcf (last accessed May 9, 2021) (De Maio et al. 2020a; Turakhia et al. 2020a), were also not considered here. We counted “possibilities” of mutations based on the reference genome: for example a noncoding C allele in the reference genome represents three possibilities for noncoding mutations (C →A, C →G, and C →U). For coding sites, we split synonymous, nonsynonymous, and nonsense mutation possibilities into separate counts. Similarly, we also used the reference genome to define the number of possible mutations within each genetic context. Sites that were masked in the alignment were still used here to define the genetic context of possible mutations at neighboring nonmasked sites.

**Fig. 1.**
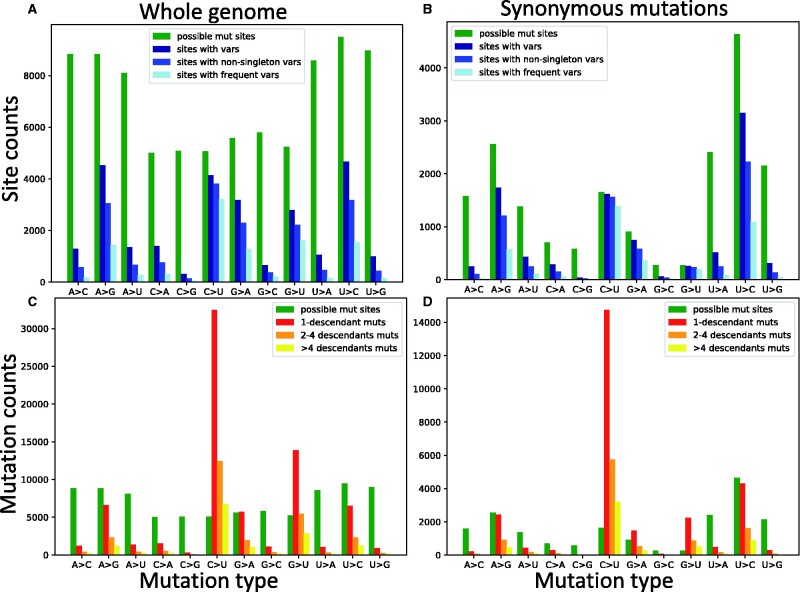
Numbers of possible mutations, observed mutations, and sites with alternative alleles. On the *X* axes are the 12 distinct types of mutation events, A →C, A →G, etc. In green, we always show the number of genome positions at which the considered mutation type is possible. In (*A*) and (*C*), we consider all possible mutations, whereas in (*B*) and (*D*), we consider only synonymous mutations. In (*A*) and (*B*) we show, on the *Y* axis, the numbers of sites with alternative alleles in the alignment (blue color hues). Note that *Y* axis scales differ among plots. In dark blue, we show the number of all sites with alternative variants of the given type; in blue, we only show the number of such sites at which the alternative variant is present in at least two sequences; in light blue, only sites at which the considered alternative allele is present in at least five sequences. By definition, in plots (*A*) and (*B*) green bars are necessarily taller than all blue ones. In (*C*) and (*D*) we show, in red, orange and yellow, the numbers of mutation events inferred with parsimony on our phylogeny. In red we show the number of mutation events of the considered type with exactly one descendant; in orange the number of these mutations with at least two but less than five descendants; in yellow, those with at least five descendants. Mutation possibilities (green) can be fewer than inferred mutations events (red, orange and yellow in plots *C* and *D*) for certain types of mutations since the same mutation event can be inferred multiple times at the same site in different parts of the phylogenetic tree.

We then inferred a mutational history with parsimony over our global maximum-likelihood tree, as described in Turakhia et al. (2020a). Given the very short branches of the SARS-CoV-2 phylogeny (median 0 and mean 0.46 mutations per phylogenetic branch per genome) uncertainty in ancestral state reconstruction conditional on the phylogenetic tree is negligible. The software for this analysis is available from https://github.com/yatisht/strain_phylogenetics (last accessed May 9, 2021). Given this inferred mutational history, for each site *x* we count all the mutation events $N_{x}^{j,d}$ inferred from the reference allele *r_x_* at position *x* to any alternative allele $j\neq r_{x}$, and with *i* descendants, excluding those that are descendants of further downstream mutations at the same site. Note that we do not count mutation events that modify a nonreference allele. For example, if at a U position of the genome we have a U →C mutation, and in one of the descendants of this mutation event we have a C →A mutation at the exact same position, then we count the first U →C mutation but not the second C →A; this is done to aid the normalization of mutation counts, see below. The inferred mutation events excluded this way are 2,040 out of a total 116,945 (1.7% of the total). At each site and for each mutation type, we kept counts of three different classes of mutations: 1) those with only one descendant tip (“singletons”); 2) those with ≥2 and ≤4 descendants (“2–4 descendants”); and 3) those with >4 descendants (“>4 descendants”). This is done to help rule out the possibility that low-frequency sequence errors might cause many artefactual mutations and bias our rate inference; in fact, such artefactual mutations are expected to tendentially have only very few descendants. Sites with frequent sequencing errors are already addressed by masking the corresponding alignment columns, following De Maio et al. (2020a) and Turakhia et al. (2020a). Numbers of mutations and mutation possibilities observed across the SARS-CoV-2 genome are given in figure 1 and supplementary figure S1, Supplementary Material online. Note that the number of observed mutations is often higher than the number of mutation possibilities. For example, a reference 4-fold degenerate site with a reference A allele counts as one A →C, one A →G, and one A →U synonymous mutation possibility. However, an A →C mutation event can occur multiple times along the phylogeny at this site, possibly resulting in multiple A →C synonymous mutations observed. When classifying mutation events into different categories, we assume that mutations happen in the genetic background of the reference genome. This might be inaccurate in some cases, but given the overall low level of divergence the effect of this approximation is expected to be very limited. In fact, given that genomes in our data set have on average about 11.9 differences from the reference, and, for simplicity, assuming that these changes are uniformly distributed across the genome and across samples, then the probability that any of two sites neighboring a mutation event (e.g., those determining if the mutation is synonymous) is different from the reference (and therefore that the reference context misrepresents the mutation context) is about 0.08%. This is likely to be an overestimate since mutation events happen on internal branches of the tree, which are on average more similar to the reference genome than the sampled genomes themselves, and so will have an average number of differences from the reference genome below the values used here of 11.9. To estimate relative mutation rates, we divide the number of mutations inferred for a certain class by the number of its mutation possibilities. For example, the relative rate of synonymous mutations $r_{a,b}$ from allele *a* to allele *b* with more than four descendants is estimated as:

(1) $$\frac{\sum_{x\in \{y|r_{y}=a\}}^{}\sum_{d>4}^{}I(x,b)N_{x}^{b,d}}{\sum_{x\in \{y|r_{y}=a\}}^{}I(x,b)},$$

where $\left\{y|r_{y}=a\right\}$ is the set of genome positions where the reference allele is *a*, and where *I*(*x*, *b*) is an indicator function that returns 1 if changing the reference at position *x* into allele *b* is a synonymous mutation, and 0 otherwise.

The low mutation rate observed in SARS-CoV-2, and in particular the low number of mutation events per phylogenetic branch, makes parsimony an efficient and reliable approach to infer mutational histories (Turakhia et al. 2020b). However, phylogenetic inference from large SARS-CoV-2 data sets is difficult due to elevated computational demand and phylogenetic uncertainty (Morel et al. 2020), and we cannot exclude the presence of errors in our phylogenetic tree, and therefore in our mutational history.

To test the effects of our approximations on the estimated mutation rates, we also used a different approach, more similar to the classical maximum likelihood inference of substitution rates in phylogenetics (see, e.g., Yang 2007), to estimate synonymous and nonsynonymous mutation rates. To do this, we used the same phylogenetic tree and inferred mutational history as above. This time, however, we count all mutations, no matter their background, as long as they don’t happen within masked regions of the alignment or from a stop codon. This removes our approximation of only considering mutation events from the reference allele. Also, this time we keep track of the context of each mutation events using the reconstructed ancestral genome at the considered mutation event, removing the assumption that the context of a mutation is the same as the reference genome at that position. Finally, when counting mutation possibilities, we consider each position in the reconstructed ancestral genomes, and weigh them using the corresponding branch lengths below them, therefore accounting for the fact that the genome evolves along the phylogeny when estimating mutation possibilities. The estimates obtained with this approach are very similar to those from our main approach described above (see supplementary fig. S4, Supplementary Material online).

Finally, we also consider another alternative approach to estimating mutation rates, based on counting the number of alignment columns at which alternative alleles are observed. We classify three classes of variant allele: 1) those present in any number of sequences; 2) those present in at least two sequences; 3) those present in at least five sequences. Numbers of different types of variable sites found in our alignment are given in figure 1 and supplementary figure S1, Supplementary Material online. This last approach is similar to those used by previous studies of SARS-CoV-2 mutation rates, and we use it for comparison with our new main approach.

## Results

### Neutral Mutation Rates in SARS-CoV-2

Here, we want to estimate the underlying mutability of different nucleotides, in a way that is as unbiased as possible with regards to how these mutations might affect the ability of the virus to replicate and spread. To do this, first, we mostly focus on synonymous mutations, since a synonymous mutation is expected to affect, on average, the fitness of the virus considerably less than a nonsynonymous mutation (Cuevas et al. 2012). Second, we only consider new SARS-CoV-2 mutations observed within the human host population, and ignore long-term divergence (between-species substitutions) which is expected to be more affected by selective forces (McDonald and Kreitman 1991) and more subject to the issues of rate changes (see, e.g., Panchin and Panchin 2020) and saturation (see, e.g., Duchêne et al. 2014). Although we cannot exclude that selection still affects some of the patterns observed below, for example, making some types of synonymous mutations more lethal for the virus than others, we tried to reduce these biases as much as possible as follows:

First, to put our results in the perspective of previous studies, we looked at numbers of sites with alternative alleles. When looking at patterns across the whole genome, it appears that all transitions (C →U, U →C, A →G, and G →A) are quite common, as well as G →U mutations; note however that A and U bases have more opportunities to mutate as they are more common in the genome (fig. 1*A*). If we focus only on possible synonymous mutations (which we expect to be less likely affected by strong selection), we see that C →U and G →U synonymous alternative variants are present at the vast majority of sites at which such variants are possible (fig. 1*B*). This means that sites at which synonymous C →U and G →U mutations might have occurred are possibly saturated with such mutations. In fact, using our phylogenetic approach to estimate numbers of mutation events of each type at each genome position, we clearly see that C →U and G →U are the most frequent mutations (fig. 1*C*), despite having fewer opportunities to occur due to GC content being lower than AT content. Focusing again on synonymous mutations, we can see that, although we don’t infer more overall G →U mutations than U →C or A →G ones, given the very low number of sites at which synonymous G →U mutations are possible, G →U and C →U are the synonymous mutations that occur proportionally more often (fig. 1*D*). We also observe similar patterns for noncoding, nonsynonymous, and 4-fold degenerate sites (supplementary fig. S1, Supplementary Material online). These observations suggest that the G →U and C →U underlying neutral mutation rates are considerably higher than all others.

Our results further suggest that inferring mutation rates from counting the number of sites with variants (the counts in fig. 1*A* and *B*, and as in, e.g., Rice et al. 2020; Simmonds 2020) can lead to underestimating the G →U and C →U synonymous mutation rates. This is because sites at which such mutations are possible are probably often saturated (multiple mutation events of exactly the same type have occurred along the phylogeny). However, saturation in previous studies was probably not as extreme as here since we investigate a considerably larger number of genomes (147,137 vs, e.g., 14,599 in Rice et al. 2020 and 865 in Simmonds 2020). Another difference of our approach from most previous studies (but similar to Rice et al. 2020), is that we aim to disentangle the contribution of selection acting on the amino acid sequence from the underlying mutation rates, and to do this we separate synonymous and nonsynonymous mutations. This has a considerable further effect on the inference of the G →U mutation rates, because there are only a few sites at which a G →U mutation is synonymous, and so most possible G →U mutations are probably under significant purifying selection; skews in the genetic code (and in particular the fact that the genetic code tends to be more robust to transitions than transversions; Freeland and Hurst 1998; Goldman 1993) are probably an important factor here. Furthermore, G is less frequent in the SARS-CoV-2 genome than U, in particular at 4-fold degenerate sites (sites where any 1-base mutation is synonymous) with a frequency of 6.5%, compared with frequencies of A (28.9%,) C (11.4%), and U (50.9%); this causes a further underrepresentation of observed G →U mutations relative to the underlying G →U mutation rate.

**Fig. 2.**
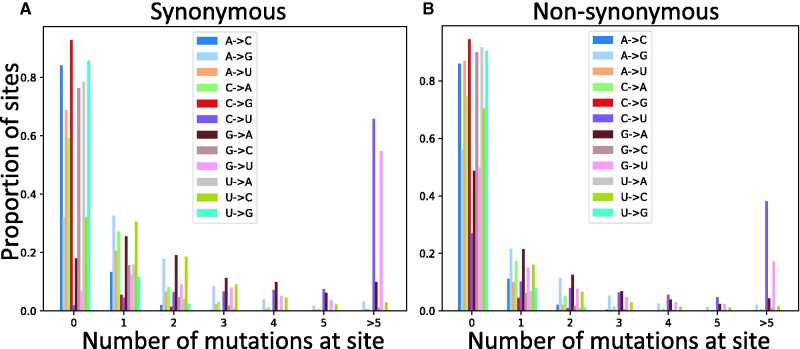
Reoccurrence of mutation events at the same sites. Proportion of sites (*Y* axis) where a given mutation (color, see legends) appears a certain number of times (*X* axis) along the phylogeny. (*A*) synonymous sites; (*B*) nonsynonymous sites.

A confirmation that many synonymous G sites are saturated with mutations can be seen in figure 2 and supplementary figure S2, Supplementary Material online. C →U and G →U mutations are by far the most homoplasic (the same mutation occurs more than once along the phylogeny), especially at synonymous and noncoding positions. This is unlikely to be the result of positive selection favoring these mutations, or of phylogenetic tree inference or parsimony mutation inference errors (since this effect is only observed in C →U and G →U mutations) and seems instead the result of underlying relatively high neutral C →U and G →U mutation rates.

We also mostly find individual C →U or G →U mutations among the most homoplasic mutations of the genome: 55 out of 59 total mutation events that occur more than 60 times at the same position are C →U or G →U. Here we ignore sites that are so homoplasic as to probably cause issues in phylogenetic inference (De Maio et al. 2020a; Turakhia et al. 2020a); these sites were masked before any analysis here, and are namely the G →U mutation at position 11083, and the C →U mutations at positions 16887 and 21575. These three sites appear as the most mutable in the genome. However, the next three most homoplasic mutations of the genome that we identify here are neither C →U nor G →U mutations. The most recurrent one is the A →G nonsynonymous (K →R) mutation at position 10323 which we inferred to have occurred 138 times, with a total of 1,187 descendants, found in 3′ →5′ context CTTAAGCTTAAGGTTGATACA. The second is a A →G nonsynonymous (K →R) mutation at position 21137, which occurred 130 times with a total of 472 descendants, found in context ATACAACAAAAGCTAGCTCTT. The third is a T →C synonymous mutation at position 27384 which occurred 119 times, with a total of 808 descendants, found in context TGGAGATTGATTAAACGAACA. We suspect that these three mutations are the results of frequent ADAR (“Adenosine Deaminase Acting on RNA”) activity, considering also their context. For example, the first two are A →G mutations with a G downstream and an A upstream (see Ko et al. 2012; Porath et al. 2014). The third site is probably affected by ADAR acting on the negative strand, since its T →C mutation is the reverse complement of the typical A →G ADAR pattern. The fourth highly homoplasic mutation we found that is not G →U or C →U is the 11th most common in the genome, a G →A nonsynonymous (G →S) mutation at position 1820, with a total of 409 descendants and in context AGCTAAAAAAGGTGCCTGGAA.

**Fig. 3.**
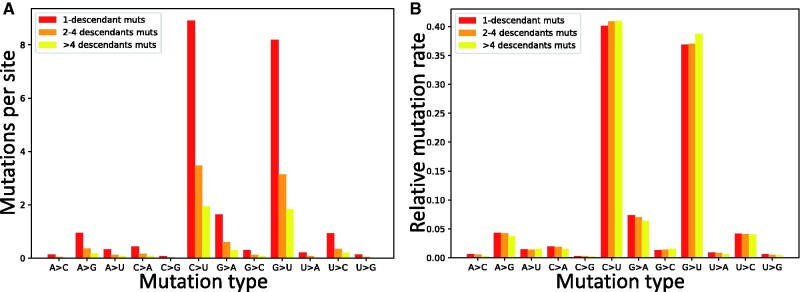
Estimated synonymous mutation rates in SARS-CoV-2. To estimate synonymous mutation rates in SARS-CoV-2, we used the counts of inferred synonymous mutation events (see fig 1*D*) normalized by the numbers of reference genome sites at which such mutations might have occurred. On the *X* axis are the 12 distinct types of mutation events, A →C, A →G, etc. In red, orange and yellow we show respectively rates obtained from counts of mutation events with one descendant, more than one but less than five descendant, and five or more descendants. (*A*) Mutation rates represented as average numbers of mutation events inferred per site at which such mutation type is possible. (*B*) Relative mutation rates (the sum of all bars of one specific color is 1.0).

One of the principal aims of our work is to estimate mutation rates while controlling the effect of selection acting on the amino acid sequence, and trying to account as much as possible for other issues such as homoplasic mutations. To do this, first, we focus on synonymous mutations, which are expected to be less subject to selective constraints than nonsynonymous ones, whereas being much more abundant than noncoding mutations. Second, we use inferred counts of mutation events (using parsimony inference along our phylogenetic tree, see Materials and Methods); this accounts for the saturation of mutation events at more mutable positions, as discussed before. Third, we separate mutation counts according to the number of observed descendants of each mutation—this allows us to have independent estimates, and to have estimates that do not rely on inferred mutation events with one descendant, which might be enriched in sequencing errors or RNA degradation (in the case errors RNA degradation would be present in our alignment). Fourth, we normalize mutation counts by the number of mutation possibilities (see eq. 1), so to account for the fact that certain mutation types (e.g., synonymous G →U mutations) are possible at fewer sites than other mutation types. For this last point, we divide the number of inferred mutations of a certain type (e.g., red, orange and yellow bars in fig. 1*D*) by the number of sites at which such mutations are possible (e.g., green bars in fig. 1*D*), always considering the reference genome as the mutational ancestral background. We find that the G →U transversion mutation rate is similar to C →U transition rate (fig. 3 and supplementary fig. S3, Supplementary Material online), and that they are both considerably higher than all other rates (about four times higher than the next highest rate, G →A, see table 1). We confirm that all other transitions, U →C, G →A, and A →G, have lower rates, but higher than all remaining transversions. In particular, the mutational process seems highly asymmetrical and strand asymmetrical, with mutation rate G →U being 82.0 times higher than U →G and 21.1 times higher than C →A (table 1). This strand asymmetry is likely the result of ROS (“reactive oxygen species”) and APOBEC activity on single-strand RNA (Graudenzi et al. 2021; Mourier et al. 2021).

**Table 1.** Mutation Rates Estimated from 4-Fold Degenerate Sites

	To A	To C	To G	To U
From A		0.039	0.310	0.123
From C	0.140		0.022	3.028
From G	0.747	0.113		2.953
From U	0.056	0.261	0.036	

Note.—Only mutation events with more than one descendant have been considered here. Rates have been normalized as typically done in phylogenetics, the normalizing constant being the sum of the rates multiplied by the frequency of the ancestral allele: $\sum_{a,b}q_{a,b}\pi (a)$, where *a*, *b* are nucleotides, $q_{a,b}$ is the original unnormalized rate from allele *a* to *b*, and π(a) is the frequency of *a* at 4-fold degenerate sites.

Our results differ markedly from those studies that either estimated mutation rates by comparing numbers of sites with alternative alleles, did not divide these counts by the numbers of opportunities for such mutations, and/or did not control for the biases in the genetic code by separating synonymous and nonsynonymous mutations (e.g., Kosuge et al. 2020; Wang et al. 2020). Our results are instead more consistent with those of studies that did take some of these steps and highlighted similarly high C →U and G →U mutation rates in SARS-CoV-2 (e.g., Rice et al. 2020). When we use an approach more similar to classical maximum-likelihood substitution rate inference (e.g., Yang 2007, see Estimation of Mutation Rates section), we find almost the same results (supplementary fig. S4, Supplementary Material online), suggesting that our approximations have little effect on our inferred rates. Although elevated C →U mutation rate in SARS-CoV-2 has been frequently observed and usually attributed to the effects of APOBEC activity, the elevated G →U has been discussed much less, but it has been usually suggested to be the result of the activity of ROS, see Rice et al. (2020) and Mourier et al. (2021). The number of descendants of mutation events seems to have little impact on our inferred relative mutation rates (fig. 3*B*); this suggests that phenomena like RNA degradation or sequencing errors, which are expected to overwhelmingly result in inferred mutation events with one descendant, do not considerably affect our mutation rate estimates.

### Context Dependencies

**Fig. 4.**
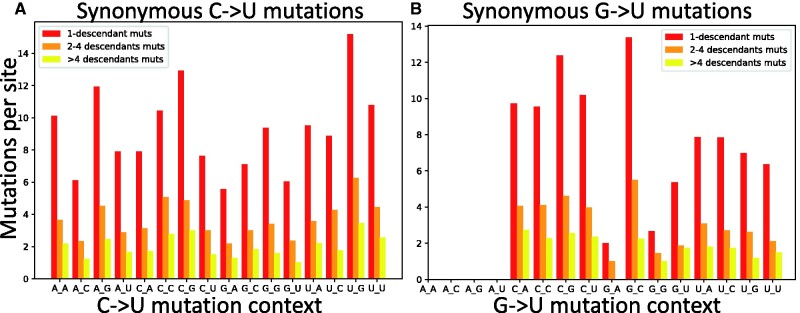
C →U and G →U synonymous mutation rates in different base contexts. Here mutation rates are calculated as in figure 3*A*. (*A*) C →U mutations. (B) G →U mutations. The *X* axis shows the context of the considered mutation (e.g., in *A*, A_G represents the trinucleotide ACG and its synonymous mutation rate into trinucleotide AUG). Colors are as in figure 3.

One of the observations that suggests APOBEC activity being the leading cause of the elevated C →U mutation rate in SARS-CoV-2 is that nucleotide context seems to affect the C →U mutation rate in a way that is consistent with the action of some of the human APOBECs (Mourier et al. 2021). Here we study the effect of neighboring base context on C →U mutability. We divide the set of possible and observed C →U mutations in classes based on the nucleotide context (5′ to 3′ preceding and following base). Even though we focus only on C →U mutations, it is still important to separate synonymous and nonsynonymous mutations, since different contexts can lead to synonymous or nonsynonymous C →U mutations with different proportions. Additionally, different contexts can be present with different frequencies in the SARS-CoV-2 genome. Focusing on synonymous mutations, and using the normalization procedure outlined above, we find that UCG →UUG appears to be the most mutable context (fig. 4*A* and supplementary fig. S5, Supplementary Material online). Note that this pattern is not observed if one considers unnormalized mutation counts, particularly those from nonsynonymous mutations (supplementary fig. S6, Supplementary Material online).

This confirms previous results which suggested an elevated UCG →UUG mutation rate (Kosuge et al. 2020; Rice et al. 2020; Sadykov et al. 2020), attributed to either the context specificity of APOBEC mutational targets, or to selection against CpG dinucleotides (Mourier et al. 2021). We discuss selection in the next section. Here, we note that although we found a signal of context affecting mutation rates in SARS-CoV-2, and although at least part of this observation is consistent with some of the known sequence targets of APOBEC, overall, C →U mutations occurring in UCG or more generally UC →UU context represent only part (and a very small part for UCG) of all C →U mutations in SARS-CoV-2 (supplementary fig. S1, Supplementary Material online) and contribute to only a small part of the C →U relative hypermutability.

Regarding long-range sequence context (nearest five bases in each direction), we observe that U in the two bases preceding a C seems to have a C →U mutagenic effect, whereas G in the previous two bases and C in the following two bases seem to reduce the mutation rate (supplementary fig. S7, Supplementary Material online).

Given the relatively high G →U mutation rate, we performed a similar analysis of context effects on G →U mutations. However, despite the elevated mutation rate, G →U synonymous mutations are quite rare (supplementary fig. S8, Supplementary Material online) and so estimates of context-dependent rates are expected to have substantial uncertainty. Additionally, some contexts are not possible for G →U synonymous mutations, for example, AG →AU mutations are never synonymous (supplementary fig. S8, Supplementary Material online). The effect of sequence context on G →U mutation rate appears overall limited, although a G being preceded or followed by a C (CG and GC contexts) seems to have a higher G →U mutation rate (fig. 4*B* and supplementary fig. S9, Supplementary Material online).

### Testing for Selection at Synonymous Sites Using Mutation Frequencies

Previous authors have discussed the effect of selection on synonymous mutations in SARS-CoV-2. For example, the elevated mutation rate in CpG context has been interpreted as a sign of selection against CpG content in SARS-CoV-2 in order to avoid zinc finger antiviral proteins (ZAP), see Mourier et al. (2021). This is consistent with similar evidence found from depleted CpG content in other coronaviruses (Woo et al. 2007). However, evidence from CpG content and substitution rates can be confounded by skewed mutation rates and mutational contexts like the ones due to APOBEC activity, which might be the leading cause of mutation in SARS-COV-2.

To overcome this limitation, and in order to disentangle the contribution of mutation and selection over the synonymous evolution of SARS-CoV-2, Rice et al. (2020) compared equilibrium frequencies inferred from SARS-CoV-2 mutation rates to observed nucleotide and dinucleotide frequencies. Since selection tends to raise the frequency of favorable alleles in a population, and to decrease the frequency of deleterious alleles, if a certain nucleotide is advantageous over another at synonymous sites, for example, if C is advantageous over U, it is expected that relatively more U →C mutations will reach fixation (completely replace the ancestral allele in the population) than C →U mutations. This means that, in the long term, there would be fewer U nucleotides in the genome than expected based on the mutation rates estimated from observed genetic variation within the population. Rice et al. (2020) observed that U nucleotides are less common at 4-fold degenerate sites in SARS-CoV-2 than expected from SARS-CoV-2 genetic variation within humans, and concluded that there is ongoing selection against U nucleotides in SARS-CoV-2. With a similar analysis, they also concluded that there is no ongoing selection against CpG content in SARS-CoV-2, despite opposite prior expectations (see Woo et al. 2007; Mourier et al. 2021).

Rice et al. (2020) assumed that the evolutionary process is stationary, that is, that mutation rates and selective pressures did not change recently (e.g., since the introduction of SARS-CoV-2 into humans). This assumption is however debatable. Due to the recent host shift and to the fact that many mutations in SARS-CoV-2 seem to be the consequence of host immune system activity, a recent significant change in mutation rates and selective pressure in SARS-CoV-2 (associated with its introduction to humans) is likely. In fact, studies have suggested that the current G → U mutation rate of SARS-CoV-2 in humans, for example, is much higher than in its reservoir hosts (Panchin and Panchin 2020; Sapoval et al. 2021). Specifically, Panchin and Panchin (2020) estimated that the G → U mutation rate in SARS-CoV-2 increased 9-fold with its introduction in humans, possibly due to difference in ROS activity between hosts.

The U content at 4-fold degenerate sites in the SARS-CoV-2 reference genome is 50.9%. This value is expected to represent very closely the U content at 4-fold degenerate sites just before the introduction of the virus to the human populations. The approach of Rice et al. (2020) consists of comparing this value with the equilibrium U content at 4-fold degenerate sites estimated from mutation rates inferred from variant sites within the human host population; a higher equilibrium U content is interpreted as evidence of selection, on average, against U content. To highlight some of the issues with this approach, we consider how a shift in mutation rates associated with the human host might affect equilibrium U content. We consider four different sets of mutation rates, each of which leads to different equilibrium nucleotide frequencies (which we estimated using the python package discreteMarkovChain v0.22 https://pypi.org/project/discreteMarkovChain/, last accessed May 9, 2021). First, we consider the mutation rates at 4-fold degenerate sites estimated following Rice et al. (2020) on our data set (inferred from the numbers of sites with variant alleles, as in fig. 1*B*); second, we use the same rates but decreasing the G →U mutation rate 9-fold (to mimic the putative reservoir mutation rate estimated in Panchin and Panchin 2020); third, we use our estimates of mutation rates as in table 1; lastly, we use the rates as in table 1 but again decreasing the G →U mutation rate 9-fold. Using these four sets of mutation rates we obtain equilibrium U content at 4-fold degenerate sites of respectively 65.5%, 52.6%, 77.4%, and 64.9%. Therefore, it is clear that changes in mutation rates associated with host shift can have dramatic effects on equilibrium frequencies, which can affect the inference of selective pressure using this approach. Furthermore, this analysis is based on the assumption that selective pressures on U content are the same in humans and in the reservoir host, which is not obvious.

To address these issues, we propose an alternative approach to test for selection acting on synonymous mutations. Since selection increases the frequency of favorable alleles and decreases the frequency of deleterious alleles within a population, we expect selection to change the relative proportion of alleles at different population frequencies. The McDonald–Kreitman test (McDonald and Kreitman 1991), for example, compares numbers of synonymous and nonsynonymous within-species polymorphisms and between-species differences; within this framework, purifying selection is expected to decrease the number of nonsynonymous between-species substitutions more than the number of nonsynonymous within-species polymorphisms.

**Fig. 5.**
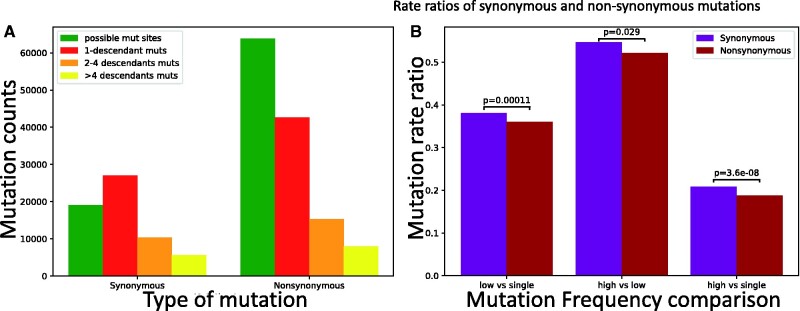
Evidence of selection affecting the population frequency of synonymous versus nonsynonymous mutations. Counts and rate ratios of SARS-CoV-2 synonymous and nonsynonymous mutations at different frequencies in the human population. (*A*) Counts of possible mutations (green), singleton mutations (red), mutations with >1 and ≤4 descendants (orange), and mutations with >4 descendants (yellow). (*B*) Ratios of higher versus lower frequency mutation rates. In the absence of selection, ratios should not be significantly different between the classes of synonymous and nonsynonymous mutations. Instead, we measure a significant deviation in each comparison, with nonsynonymous mutations being relatively depleted of high frequency mutations. We calculated *P* values using the chi2_contingency function of the Scipy.stats package (Virtanen et al. 2020). On the *X* axis, “single” refers to mutations with one descendant, “low” to mutations with 2–4 descendants, and “high” to mutations with >4 descendants. For example, “high versus single” refers to the comparison of rate of mutations with >4 descendants versus the rate of mutations with one descendant.

However, the emergence of SARS-CoV-2 in humans is relatively recent, too recent for variants (and especially a statistically sufficient number of variants) to have reached fixation within the human population, making this type of test inapplicable. Instead, we use an alternative version in which we compare low-frequency mutations against high-frequency ones. Although our aim is to focus on comparing different types of synonymous mutations, we first apply this approach to compare synonymous and nonsynonymous mutations, so as to make the similarity to the McDonald–Kreitman test more apparent. Nonsynonymous mutations in SARS-CoV-2 appear significantly shifted toward lower frequencies (fig. 5), consistent with the expectation that purifying selection tends to decrease the frequencies of nonsynonymous mutations more than the frequencies of synonymous mutations.

**Fig. 6.**
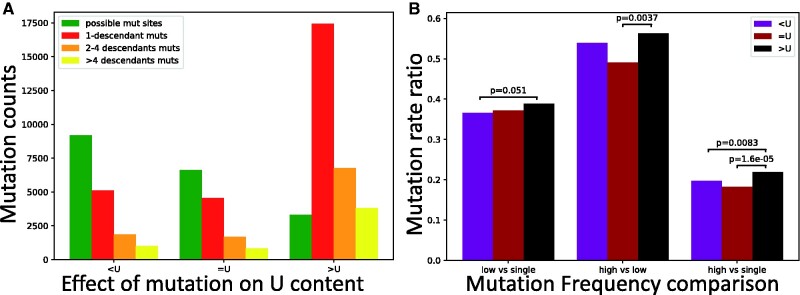
Test of selection affecting U content at synonymous sites. Values are the same as in figure 5, but this time we focus on synonymous mutations that decrease U content (“<U”), increase it (“>U”), or leave it unaltered (“=U”). Only *P* values below 0.1 are shown.

Next, we focused on the hypothesis that U variants at synonymous sites are on average mildly deleterious (Rice et al. 2020). This time, not all of our comparisons are significant, but those that are, consistently suggest that selection favors U variants (fig. 6), not the opposite. As we discussed before, the difference with the estimate from Rice et al. (2020) probably lies in the assumption of stationarity in SARS-CoV-2 evolution (although we also used a larger data set and a different approach to estimate mutation rates). It’s hard to draw clear conclusions regarding individual genes, as, expectedly, we lose statistical power when analyzing each gene individually, in particular for the shorter genes (supplementary figs. S10 and S11, Supplementary Material online).

A possible explanation for the pattern we observe is that many mutations increasing U content are C →U or G →U, and therefore may sometimes reduce CpG content. Selection against CpG content has already been suggested in coronaviruses (Woo et al. 2007), and is expected due to ZAP activity (Mourier et al. 2021). However, using our approach the evidence in support of this hypothesis is not significant (supplementary fig. S12, Supplementary Material online). Similarly, when investigating possible selection affecting GC content, only one of the comparisons is significant (supplementary fig. S13, Supplementary Material online).

## Discussion

In this study, we investigated mutation rates and selection at synonymous sites in SARS-CoV-2. We used a different methodology than previous studies, so as to exploit more than 147,000 SARS-CoV-2 genome sequences while trying to avoid inaccuracies due to mutation saturation. Our approach also controls for the fact that different types of mutations can have, on average, different fitness due to their impact on the amino acid sequence and due to skews in the genetic code. In fact, we focus on synonymous mutations, which are expected to be subject to lower selective pressure than nonsynonymus mutations. Also, we did not assume equilibrium in SARS-CoV-2 genome evolution, which is unlikely given the recent shift of host the virus underwent. We also investigated some possible selective pressure affecting synonymus mutations.

We found that two mutation rates, C →U and G →U, are similar to each other and much higher than all others mutation rates, leading to extremely frequent homoplasies. This also means that the SARS-CoV-2 mutation process is very far from being symmetric or strand symmetric; it is also considerably far from equilibrium. We found some sites with extremely high non-C →U non-G →U mutation rates that are consistent with targeted ADAR activity.

A consequence of these findings is that popular and efficient phylogenetic substitution models such as JC69 (Jukes et al. 1969), HKY85 (Hasegawa et al. 1985), or GY94 (Goldman and Yang 1994) might be inappropriate with SARS-CoV-2 data and might cause biases in the inference of phylogenetic trees (Turakhia et al. 2020a), selection (van Dorp et al. 2020a; Graudenzi et al. 2021), and recombination (Yi 2020) due to the neutral high recurrence of certain mutations. It is therefore probably important to adopt phylogenetic substitution model that can account for elevated C →U and G →U rates, such as the nonreversible UNREST model (Yang 1994), or otherwise to control for the skews in the mutational process (see, e.g., Graudenzi et al. 2021). In our experience, we have found the UNREST model to often cause numerical instability in current phylogenetic packages that allow it (data not shown here). In the future, if we want to alleviate these biases, it will be important to implement such more general substitution models more broadly and in a more numerically stable way.

When investigating selection at synonymous sites possibly affecting CpG or GC content, we found mostly nonsignificant patterns. We also investigated the possibility of selection against U content in SARS-CoV-2 (Rice et al. 2020), but found evidence in the opposite direction. Although further analyses will be needed to establish with confidence what kind of selection acts on SARS-CoV-2 synonymous mutations, we suggest that inference based on assumption of genome equilibrium can be biased by changes in mutation rates and selective pressures associated with host shift. Although we tried to account for possible biases as much as possible, our methods still have some limitations. First of all, our inference of mutation events is based on a prior phylogenetic inference, but tree inference from SARS-CoV-2 data can be unreliable, in particular regarding low-level branches of the phylogeny (those closer to the samples and further from the root), in part due to the low genetic diversity among sequences, but more importantly due to highly homoplasic mutations and sequencing errors (Morel et al. 2020; Turakhia et al. 2020a); Removing extremely homoplasic sites and putative errors can be of help, but, as we have shown, the mutational spectrum in SARS-CoV-2 is highly skewed, and so removing all homoplasic sites would result in removing most C →U and G →U synonymous mutations, which in turn would cause the loss of a large part of the available phylogenetic signal. As mentioned above, our phylogenetic inference might also have been negatively affected by the choice of substitution models; however, currently, more realistic models like UNREST are either not implemented or are numerically unstable in sufficiently efficient phylogenetic packages such as Minh et al. (2020), Price et al. (2010), and Kozlov et al. (2019). In this study, we tried not to rely excessively on individual inferences of mutation events, but rather focused on general patterns averaged over many sites and clades, which we think should provide robust inference despite the fact the inference of individual mutation events might not be reliable. However, a potential bias that might affect our result derives from the fact that some sites are very homoplasic, and our phylogenetic inference might lead to an over-parsimonious inference of their mutational history. This, in turn, might lead us to underestimate their mutation rate and overestimate their number of descendant tips per mutation events. In the future, a Bayesian phylogenetic approach might be useful to assess and possibly resolve this issue and assess its impact on our inference of selective pressure; however so far Bayesian phylogenetic inference has proved prohibitive with data sets of this size.

To further investigate and disentangle selective and mutational forces in SARS-CoV-2, it would be very promising to combine an analysis of between-patients and within-patient SARS-CoV-2 genetic variation. In a similar way as selection is expected to decrease the frequency of deleterious SARS-CoV-2 alleles at the human population level, the same is true at the within-host levels, as selection is expected to act on within-host deleterious mutations and often prevent them from reaching high frequency and transmit further on. Although within-patient genetic diversity data can indeed be very informative of the SARS-CoV-2 evolutionary patterns (Di Giorgio et al. 2020; Kuipers et al. 2020; Graudenzi et al. 2021), it is important to consider that such data is also more prone to sequencing, read processing, and RNA degradation issues. These issues cause some errors in consensus sequences (De Maio et al. 2020a; Turakhia et al. 2020a) but they are expected to be even more problematic at the level of detected within-host variation. Indeed, so far there is reason to be cautious when interpreting these data (De Maio et al. 2020a), especially with specific data sets (see https://virological.org/t/gained-stops-in-data-from-the-peter-doherty-institute-for-infection-and-immunity/486, last accessed May 9, 2021). Given reliable data regarding evolution at different levels (e.g., within a patient, between patients, and between hosts), it would be interesting to combine these sources of information to improve estimates of selective pressures (see, e.g., Wilson et al. 2011; De Maio, Schlötterer, et al. 2013b).

Our methodology to detect selection could also be improved by using the full site frequency spectrum of mutations, instead of categorizing mutations into frequency classes (see, e.g., Zeng and Charlesworth 2009; Clemente and Vogl 2012). However, it is important to consider that complex epidemiological dynamics and sampling biases in SARS-CoV-2 mean that it is hard to interpret the shape of any individual site frequency spectrum in terms of the effects of mutation and selection. We infer mutation and selection patterns by comparing properties of site frequency spectra associated with different mutation types. This approach should be robust to the effects of variable population dynamics and sampling biases, since these forces are expected to affect in the same way site frequency spectra associated with different mutation types.

Although we only applied our methods to SARS-CoV-2 genome data, the same approaches could be used for variation data from other viruses. Other studies have suggested that mutation rates in SARS-CoV-2 are quite different from those in SARS-CoV and MERS, with SARS-CoV-2 showing, for example, higher G →U rates (Panchin and Panchin 2020; Sapoval et al. 2021). It would be of interest to test if these estimates would change after controlling for saturation and selection at the amino acid level.

In the future, we hope to extend our current approach to also study insertions and deletions (indels): their frequency, recurrence, and possible fitness effects (see, e.g., Kemp et al. 2020). However, we expect that incorporating indels in our approach will be challenging since standard phylogenetic approaches rarely model indels.

## Supplementary Material

evab087_Supplementary_Data
