# Supplementary material for: Mutation Rates and Selection on Synonymous Mutations in SARS-CoV-2
Source: Genome Biol Evol. 2021 Apr 24;13(5):evab087. doi: 10.1093/gbe/evab087 (PMC8135539; doi:10.1093/gbe/evab087)
Supplement: evab087_Supplementary_Data [file evab087_supplementary_data.zip › Supplement.pdf]

Supplementary Material for: Mutation rates and selection on synonymous mutations in SARS-CoV-2

Nicola De Maio,[*,1] Conor R. Walker,[1,2] Yatish Turakhia,[3,4] Robert Lanfear,[5] Russell Corbett-Detig,[3,4] and Nick Goldman[1]

[1]European Molecular Biology Laboratory, European Bioinformatics Institute, Wellcome Genome Campus, Hinxton, Cambridgeshire, CB10 1SD, UK
[2] Department of Genetics, University of Cambridge, Cambridge, CB2 3EH, UK
[3] Department of Biomolecular Engineering, University of California Santa Cruz, Santa Cruz, CA 95064, USA
[4] Genomics Institute, University of California Santa Cruz, Santa Cruz, CA 95064, USA
[5] Department of Ecology and Evolution, Research School of Biology, Australian National University, Canberra, ACT 2601, Australia
[*] Email to: demaio@ebi.ac.uk

Supplementary Figures

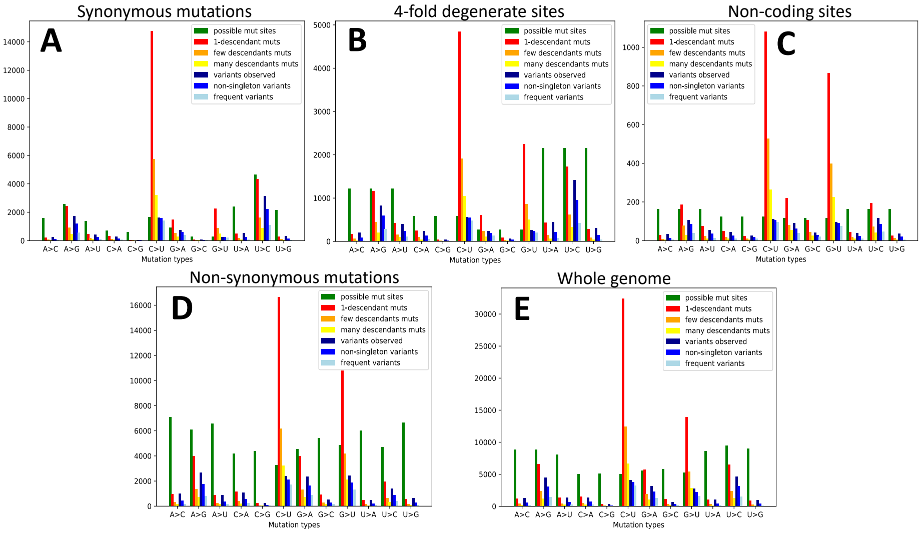

Figure S1: **Numbers of possible mutations, observed mutations, and variable sites in the SARS-CoV-2 genome**. Counts of mutation events for each site class: **A** synonymous sites, **B** 4-fold degenerate sites, **C** non-coding sites, **D** non-synonymous sites, **E** all sites. On the X axis are the 12 distinct types of mutation events, A→C, A→G, etc. In green we show the number of reference sites at which a mutation might have occurred. In red, orange and yellow we show respectively the number of observed mutations with 1 descendant, more than 1 but less than 5 descendants, and more than 4 descendants. In dark blue, blue, and light blue, we show respectively the number of sites with > 0, > 1, and > 4 variants of the given type.

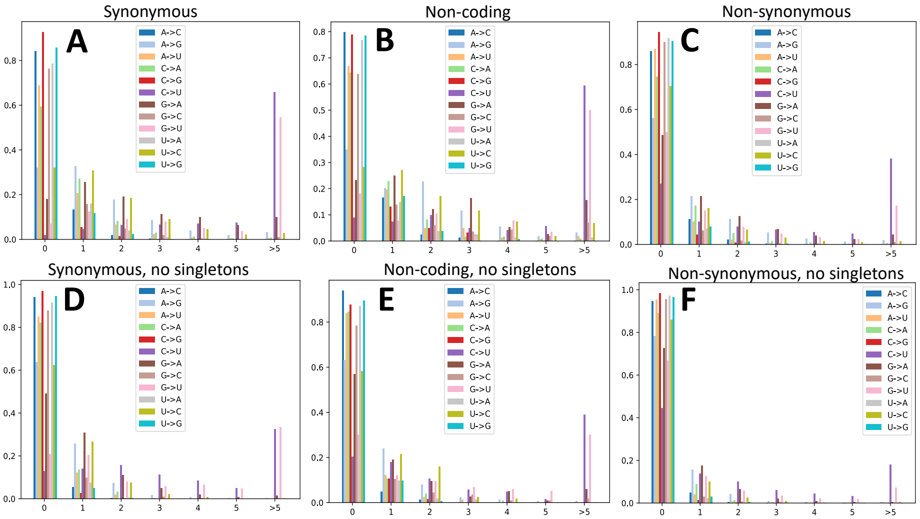

Figure S2: **Re-occurrence of mutation events at the same sites**. Here we show the proportion of sites (Y axis) where a given mutation (color, see legends) appears a certain number of times (X axis) along the phylogeny. **A** synonymous sites; **B** non-coding sites; **C** non-synonymous sites; **D** synonymous sites, but counting only mutation events with more than 1 descendant; **E** non-coding sites, only mutations with more than 1 descendant; **F** non-synonymous sites, only mutations with more than one descendant.

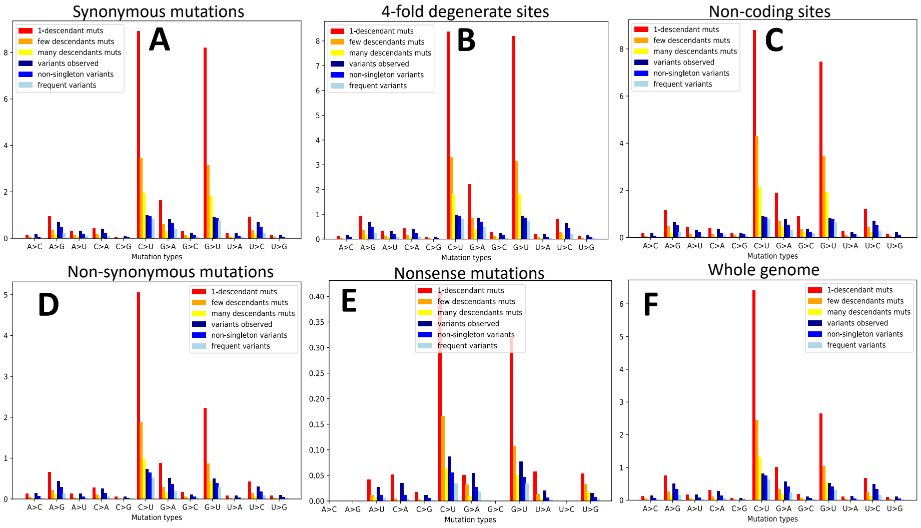

Figure S3: **Mutation rates estimated from mutation counts and variable sites counts**. On the X axis are the 12 distinct types of mutation events, A→C, A→G, etc. On the Y axis are the inferred mutation rates for **A** synonymous mutations, **B** 4-fold degenerate sites, **C** non-coding mutations, **D** non-synonymous mutations, **E** nonsense mutations, **F** all mutations. In red, orange and yellow we show respectively the mutation rates inferred from the numbers of observed mutations with 1 descendant, more than 1 but less than 5 descendant, and more than 4 descendant (and dividing each count by the number of reference sites where such mutations might have happened). In dark blue, blue, and light blue, we show respectively the mutation rates inferred from the numbers of sites with > 0, > 1, and > 4 variants of the given type.

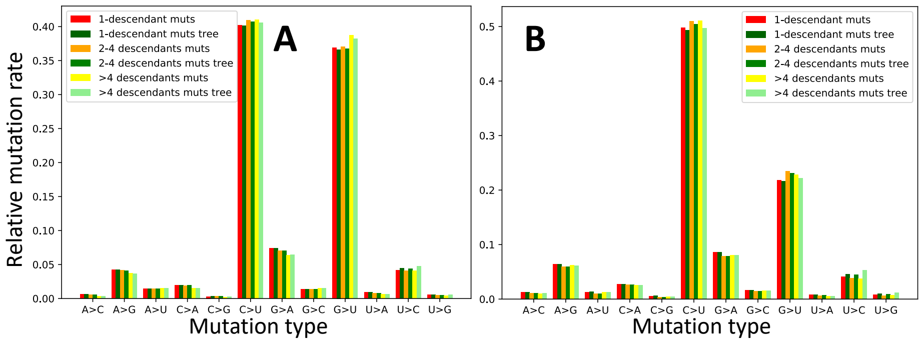

Figure S4: **Mutation rates estimated from mutation counts and variable sites counts, compared to those estimated with an approach similar to maximum likelihood**. On the X axis are the 12 distinct types of mutation events, A→C, A→G, etc. On the Y axis are the inferred normalized mutation rates (the sum of all bars of one specific color is 1.0) for **A** synonymous mutations, **B** non-synonymous mutations. In red, orange and yellow we show respectively the mutation rates inferred from the numbers of observed mutations with 1 descendant, more than 1 but less than 5 descendants, and more than 4 descendants (and dividing each count by the number of reference sites where such mutations might have happened), similar to Figure 3. In dark green, green, and light green, we show rates estimated with an approach similar to maximum likelihood rate estimation, which relaxes many of the assumptions made in our main approach (see Section 5.2). In dark green we show estimates from numbers of observed mutations with 1 descendant, in green with more than 1 but less than 5 descendants, and in light green with 5 descendants or more.

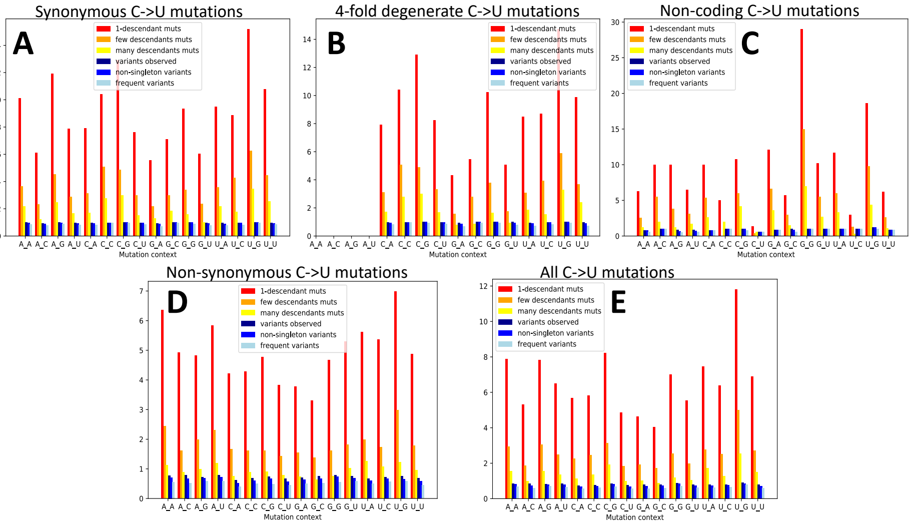

Figure S5: **C→U mutation rates in different base contexts**. C→U mutation rate depending on the previous and next base (5' and 3' base neighbours, shown on the X axis). A_G represents, for example, the trinucleotide ACG and its mutation rate into trinucleotide AUG. Colors are as in legend fig. 3. **A** synonymous sites, **B** 4-fold degenerate sites, **C** non-coding sites, **D** non-synonymous sites, **E** all sites.

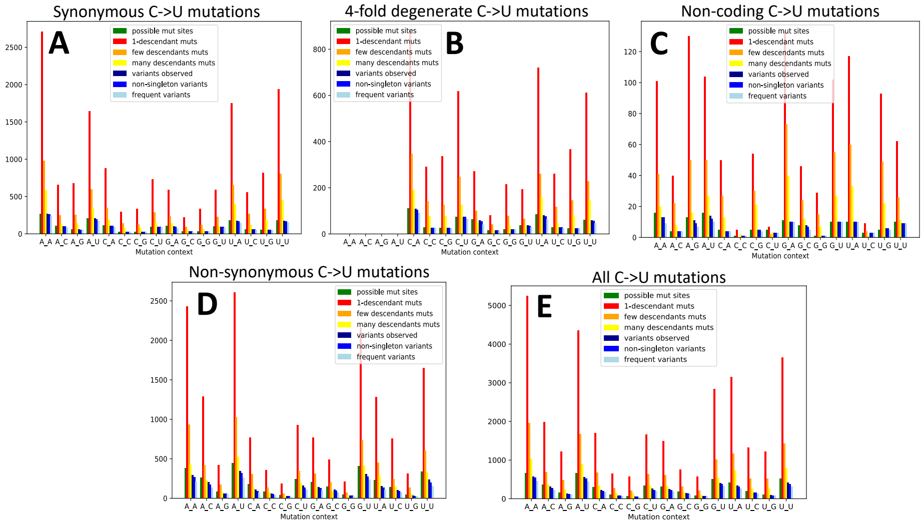

Figure S6: **C→U mutation and mutation possibility counts in different base contexts**. C→U mutation counts depending on the previous and next base (5' and 3' base neighbours, shown on the X axis). A_G represents, for example, the trinucleotide ACG and its mutation counts into trinucleotide AUG. Colors are as in legend of fig. 1. **A** synonymous sites, **B** 4-fold degenerate sites, **C** non-coding sites, **D** non-synonymous sites, **E** all sites.

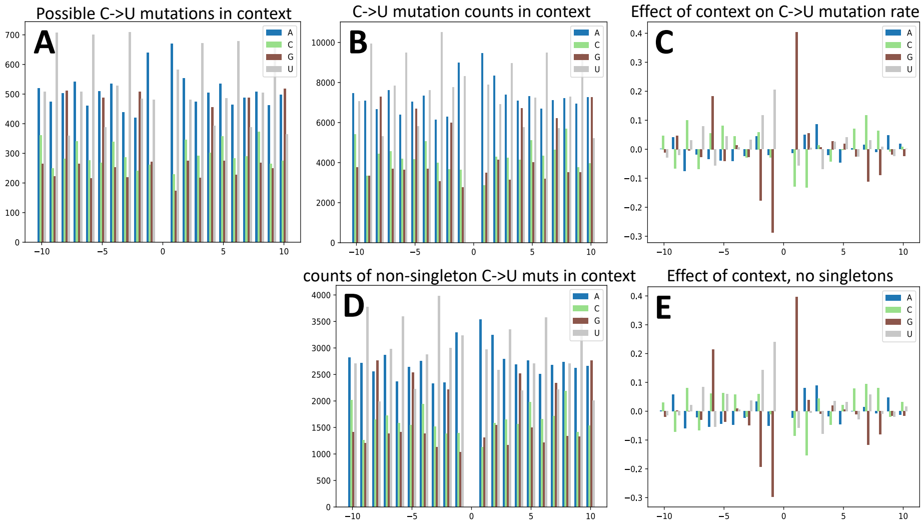

Figure S7: **C→U synonymous mutations and mutation rates in different longer-range base contexts**. Here we consider only synonymous C→U mutations. X axis values represent the distance of the considered base to the one whose mutation rate is considered. Y axis values represent **A** the numbers of possible synonymous mutations with the given context, **B** the numbers of observed synonymous mutations, **D** the numbers of observed non-singleton mutations, **C** the effect on mutation rate that the considered base at the considered position has, **E** same as **C** but without considering mutations with only one descendant. For example, the value for base G at position -1 in plot **C** represents the increase in GC→GU mutation rate vs all other C→U mutation rates; a Y axis value of 0.1 means that the given context increases the background mutation rate by 10%.

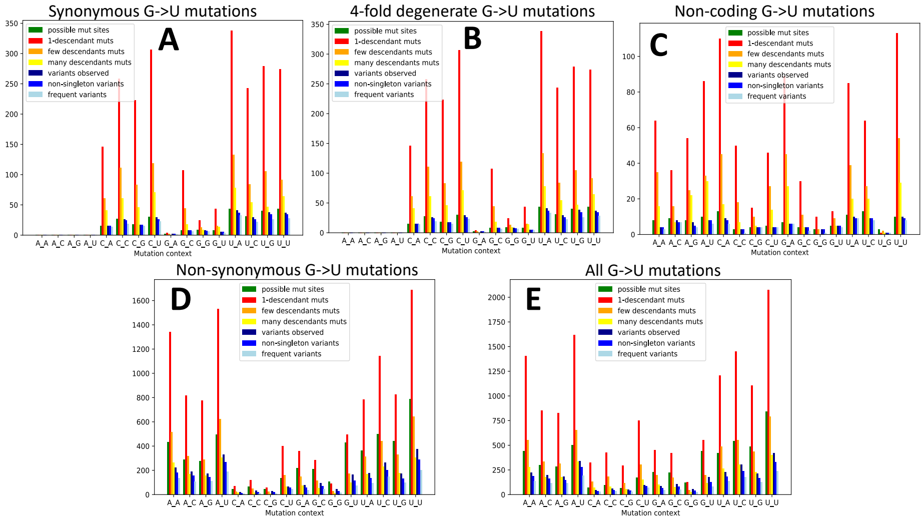

Figure S8: **G→U mutation and mutation possibility counts in different base contexts**. The X axes show the 16 types of mutation contexts for a G→U mutation, for example C_A means the rate of mutation from trinucleotide CGA to trinucleotide CUA. Colors are as in fig. 1. **A** synonymous sites, **B** 4-fold degenerate sites, **C** non-coding sites, **D** non-synonymous sites, **E** all sites.

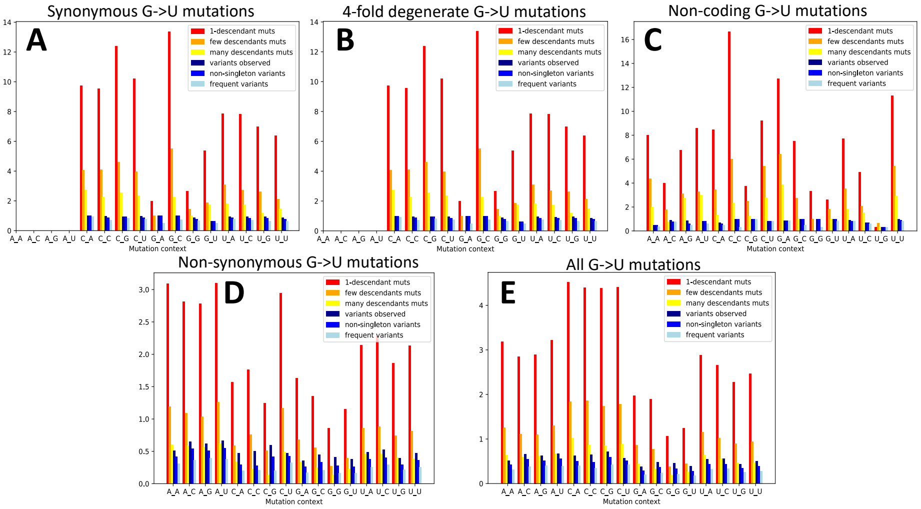

Figure S9: **G→U mutation rates in different base contexts**. G→U mutation rate depending on the previous and next base (5' and 3' base neighbours, shown on the X axis). C_A represents, for example, the trinucleotide CGA and its synonymous mutation rate into trinucleotide CUA. Colors are as in legend of fig. 3. **A** synonymous sites, **B** 4-fold degenerate sites, **C** non-coding sites, **D** non-synonymous sites, **E** all sites.

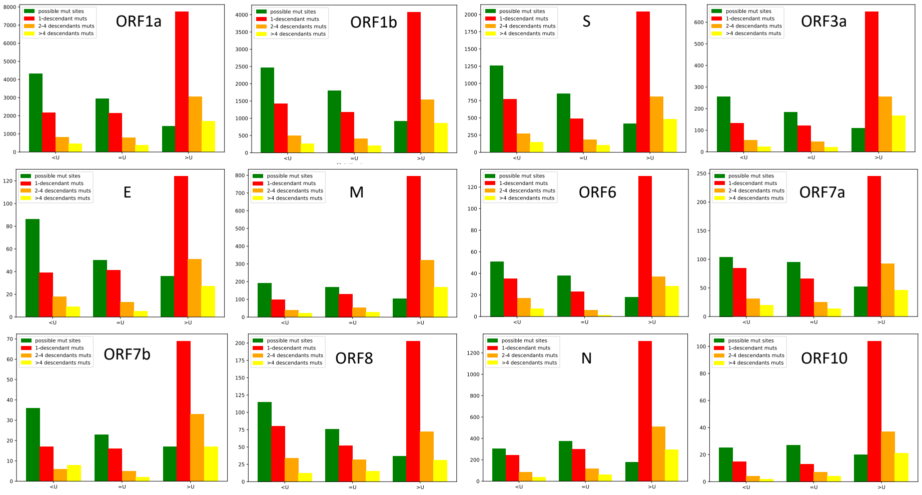

Figure S10: **Numbers of sites for testing selection on U content at synonymous sites, split by gene**. Values are the same as in fig. 6A, but they are split according to the gene - each plot refers to values for one gene.

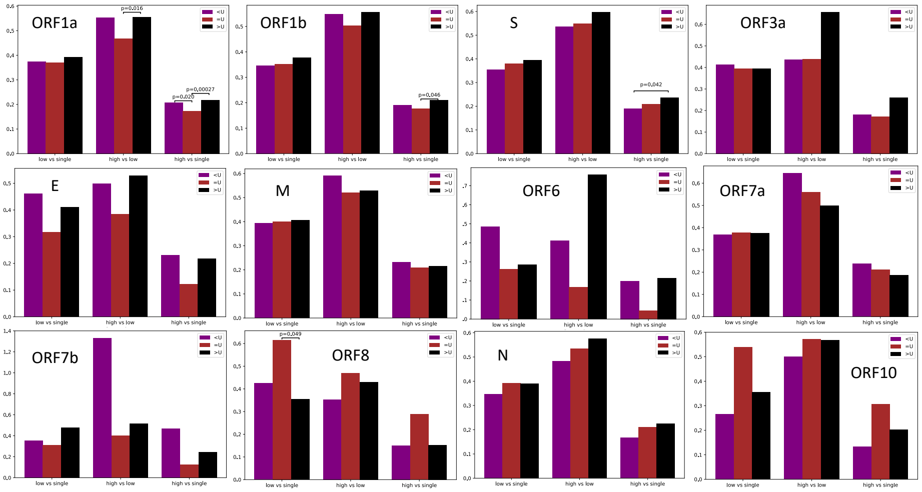

Figure S11: **Tests selection on U content at synonymous sites**. Values are the same as in fig. 6B, but here they are split according to individual genes.

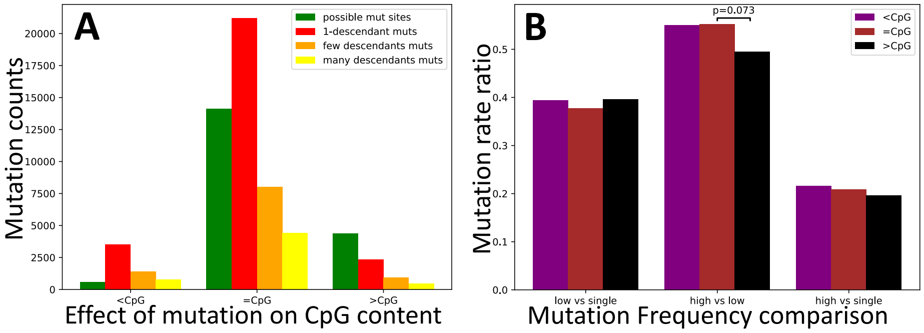

Figure S12: **Test of selection affecting CpG content at synonymous sites**. Values are the same as in fig. 5, but this time we focus on synonymous mutations that decrease CpG content ("<CpG"), increase it (">CpG"), or leave it unaltered ("=CpG"). Only p-values below 0.1 are shown.

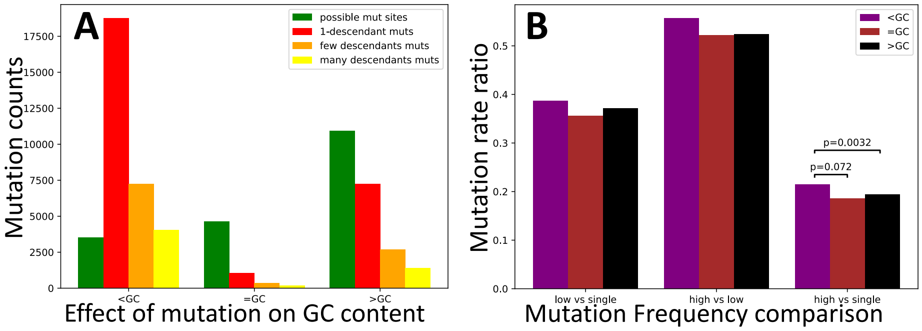

Figure S13: **Test of selection affecting GC content at synonymous sites**. Values are the same as in fig. 5, but this time we focus on synonymous mutations that decrease GC content ("<GC"), increase it (">GC"), or leave it unaltered ("=GC"). Only p-values below 0.1 are shown.
